# Bioactive Compounds of Citrus Fruits: A Review of Composition and Health Benefits of Carotenoids, Flavonoids, Limonoids, and Terpenes

**DOI:** 10.3390/antiox11020239

**Published:** 2022-01-26

**Authors:** Ramesh Kumar Saini, Arina Ranjit, Kavita Sharma, Parchuri Prasad, Xiaomin Shang, Karekal Girinur Mallikarjuna Gowda, Young-Soo Keum

**Affiliations:** 1 Department of Crop Science, Konkuk University, Seoul 143-701, Korea; saini1997@konkuk.ac.kr; 2 Biomedical and Pharmaceutical Sciences, Kasiska Division of Health Sciences, College of Pharmacy, Pocatello, ID 83209, USA; arinaranjit@isu.edu (A.R.); sharkum2@isu.edu (K.S.); 3 Institute of Biological Chemistry, Washington State University, Pullman, WA 99164, USA; prasad.parchuri@wsu.edu; 4 Jilin Provincial Key Laboratory of Nutrition and Functional Food, Jilin University, Changchun 130062, China; xmshang@jlu.edu.cn; 5 Department of Biochemistry, Padmashree Institute of Management and Sciences, Bengaluru 560060, India; mallikarjunagowda367@gmail.com

**Keywords:** orange, mandarin, polymethoxylated flavones (PMFs), nobiletin, essential oil, β-citraurin, limonene, metabolic syndrome, neurodegenerative diseases, cardiovascular disease (CVD)

## Abstract

The increased consumption of fruits, vegetables, and whole grains contributes to the reduced risk of many diseases related to metabolic syndrome, including neurodegenerative diseases, cardiovascular disease (CVD), diabetes, and cancer. Citrus, the genus *Citrus* L., is one of the most important fruit crops, rich in carotenoids, flavonoids, terpenes, limonoids, and many other bioactive compounds of nutritional and nutraceutical value. Moreover, polymethoxylated flavones (PMFs), a unique class of bioactive flavonoids, abundantly occur in citrus fruits. In addition, citrus essential oil, rich in limonoids and terpenes, is an economically important product due to its potent antioxidant, antimicrobial, and flavoring properties. Mechanistic, observational, and intervention studies have demonstrated the health benefits of citrus bioactives in minimizing the risk of metabolic syndrome. This review provides a comprehensive view of the composition of carotenoids, flavonoids, terpenes, and limonoids of citrus fruits and their associated health benefits.

## 1. Introduction

Mechanistic, observational, and intervention studies have shown that increased consumption of fruits, vegetables, and whole grains contributes to the reduced risk of many diseases related to metabolic syndrome, including neurodegenerative diseases, cardiovascular disease (CVD), type 2 diabetes, and cancer [1,2]. These diseases are primarily associated with systemic and low-grade chronic inflammation prompted by oxidative stress. The bioactive compounds present in fruits, vegetables, and whole grains prevent the oxidative damage of cells by detoxifying the free radicals, thus minimizing the incidence of such diseases [3].

Citrus, the genus *Citrus* L. of the family Rutaceae, subfamily Aurantioideae [4], is one of the most important fruit crops, including pomelo, sweet orange, sour, lemon, lime, citron, grapefruit, kumquat, and hybrids [5,6]. The citrus fruit species widely investigated for their bioactive composition and their health benefits are listed in Table 1.

Southeast Asia, especially the Yunnan province of Southwest China, Myanmar, and Northeastern India in the Himalayan foothills, is generally considered the origin of *Citrus* [4]. Citrus plants are now widely cultivated in the tropical and subtropical areas of the world, especially China, followed by Brazil and the European Union, with annual production of approximately 102 million tons [7]. Oranges account for half of the production/exports, followed by tangerines/mandarins (one third of total citrus production), lemons/limes (≈8%), and grapefruit (≈7%) [7].

The *Citrus* genus is popular worldwide because of its pleasant flavor and richness of bioactive and nutrients. Citrus fruits are an excellent source of bioactive compounds [3], mainly phenolic compounds (flavonoids, phenolic acids, and coumarins), terpenoids (limonoids and carotenoids), and pectin [5,8,9]. In addition, citrus fruits are rich in nutrients, such as ascorbic acid (vitamin C), tocopherols and tocotrienols (vitamin E), and minerals (selenium, zinc, copper, iron, and manganese) [3,5,8].

Several outstanding reviews have recently been published on citrus’ bioactive composition and health benefits (Table 2), centering primarily on flavonoids and essential oil (terpenes and limonoids). However, a comprehensive review of all major bioactive components of citrus fruits is lacking. Thus, this review provides a comprehensive view of the composition of flavonoids, carotenoids, terpenes, and limonoids of citrus fruits and their associated health benefits. Most importantly, this review highlights the significant recent advancement in this area.

## 2. Literature Search Methodology

Available electronic databases, especially Web of Science, PubMed, and Google Scholar, were searched for studies (review or experimental) that analyzed the composition of bioactive compounds in citrus fruits and their health benefits (in vitro, in vivo, and epidemiological). The primary search keywords were: (1) **Citrus** (title) and **antioxidants** (topic) or **health** (topic) and (2) **Citrus** (title) and **bioactive** (topic) or **health** (topic). The other keywords were: (1) **Citrus** (title) and **flavonoid** (title) or **health** (topic); (2) **Citrus** (title) and **carotenoids** (title) or **health** (topic); (3) **Citrus** (title) and **essential oil** (title) or **health** (topic); (4) **Citrus** (title) and **essential oil** (title) or **health** (topic); (5) **Fruits** (title) and **health** (topic); and (6) **Diet** (title) and **health** (topic). The relevant 320 articles were downloaded; they had been published mostly between 2018 and 2022. A total of 135 articles, including 128 published in the years 2022 (02), 2021 (37), 2020 (35), and 2019 (30), are discussed in this review.

## 3. Bioactive Compounds of Citrus Fruits

### 3.1. Flavonoids

**Figure 1:**
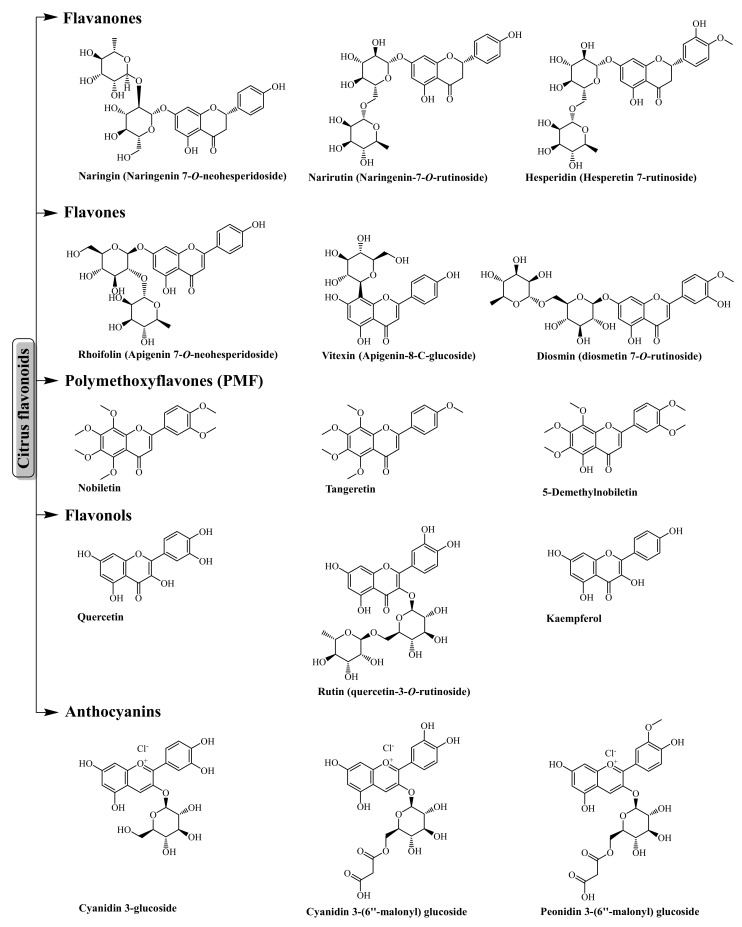
The major flavonoids of citrus fruits.

Flavonoids, a significant contributor of antioxidant components within the human diet, are a class of polyphenolic secondary metabolites widely found in plants. Chemically, flavonoids are composed of a 15-carbon skeleton (C6-C3-C6) with two six-carbon phenyl rings joined by a heterocyclic ring containing the embedded oxygen. According to the substitution patterns of a heterocyclic ring, flavonoids can be divided into subgroups, such as flavones, flavonols, flavanones, flavanonols, flavanols (flavan-3-ols), isoflavones, and anthocyanins. Citrus fruits contain a substantial amount of flavanone-7-O-glycosides (e.g., naringin, eriocitrin, hesperidin, and narirutin), flavones (e.g., rhoifolin, vitexin, diosmin), polymethoxylated flavones (PMFs, e.g., nobiletin, tangeritin, and 5-demethyl nobiletin), flavonols (quercetin, rutin, and kaempferol), and anthocyanin (cyanidin and peonidin glucosides) [28,29,30,31] (Figure 1).

PMFs are a unique class of bioactive flavonoids with more than two methoxyl (–OCH_3_) groups on their chemical skeletons, and they abundantly occur in citrus fruits [29]. PMFs have attracted growing interest in recent years due to their anti-inflammatory [32], anti-atherosclerosis [33], anti-obesity [34,35], and anti-cancer properties [36]. Moreover, demethylated PMFs, a product of fruit metabolism, chemical reactions during the drying process, and human metabolism, possess greater anticancer and anti-inflammatory activities than their corresponding methylated counterparts [37].

Deng et al. [38] isolated 11 flavonoids from (*cv*. Shatianyu) pulp; among them, naringin and rhoifolin showed the highest oxygen radical absorbance capacity (ORAC) activity. However, melitidin, bergamjuicin, and naringin were the major contributors to the ORAC activity in flavonoid extracts. In the albedo (inner layer) of ancient Mediterranean citrus fruit, flavonoids occupied 89.34% of polyphenolic fractions, dominated by flavanones eriocitrin and hesperidin as significant components, which accounted for 52.81% and 31.31% of the total flavonoids, respectively [30].

Citrus fruits contain the highest amount of flavonoids during the middle stages (60–80 days after pollination (DAP)) of development, and a decrease during complete maturation, probably due to the high expression of *Chalcone synthase-1* (*CHS-1*) and *chalcone isomerase*, the rate-limiting enzymes in flavonoid biosynthesis [28,39,40]. In contrast, hesperidin peaked at the last developmental stage in the juice sacs of lemon (*cv*. Akragas) [41]. Moreover, the citrus fruit peel flavedo (outer layer) and the albedo contain more flavonoids than the juice sacs [41]. Among the 116 citrus accessions screened by Peng et al. [28], the highest amounts of PMFs, especially OCH_3_-PMFs (nobiletin and tangeritin), were recorded in loose-skin mandarins (including mandarins and tangerines) and their hybrids, followed by tangelo (*C. reticulata* × *C. paradisi*), sweet orange, junos, Rangpur lime, sour orange, and grapefruit [28]. Interestingly, the content of nobiletin, 5-demethylnobiletin, and tangeritin increased during the maturation and reached the highest at 60 DAP and decreased again (60–210 DAP). In the Persian lime, the highest amounts of flavanones (hesperidin, 2005 µg/g; eriocitrin, 1171 µg/g; and narirutin, 1207 µg/g) and flavones (disomin, 366 µg/g; rhoifolin, 285 µg/g; and vitexin, 237 µg/g) were recorded at 12 weeks of growth and found to reduce at complete maturity (16 weeks). In contrast, in this study, the contents of flavanols (rutin and quercetin) were found to be highest at five weeks of maturation.

In a comparative study, the highest amounts of total phenolic compounds were recorded in the albedo of unripe sweet orange (*cv*. Washington navel, 10910 mg kg^−1^ DW) and accounted for 50% of the cumulative content (flavedo + albedo + juice sacs), followed by orange (*cv*. Tarocco) flavedo, lemon (*cv*. Akragas) flavedo, and pummelo (*cv*. Chandler) albedo of unripe stages [41]. In this study, in the juice sacs of ripened fruits, flavanone hesperidin was the dominating phenolic compound in lemon (2213 mg/kg DW) and oranges (1957 and 1975 mg/kg DW in Washington novel and Tarocco, respectively), whereas flavanone narirutin was the most prevalent in pummelo (292 mg/kg DW). A significant amount of flavanone eriocitrin was recorded from lemon (913 mg/kg DW).

In the fruit pulp of Sanguinello and Tarocco blood oranges, hesperidin (78–143 mg/100 g) dominated, followed by narirutine (37.0–93.0 mg/100 g) and quercetin (28.1–42 mg/100 g) [8]. In addition, in this study, cyanidin 3-(6″-malonylglucoside) and cyanidin 3-glucoside were detected in the fruit pulp.

### 3.2. Carotenoids and Apocarotenoids

**Figure 2:**
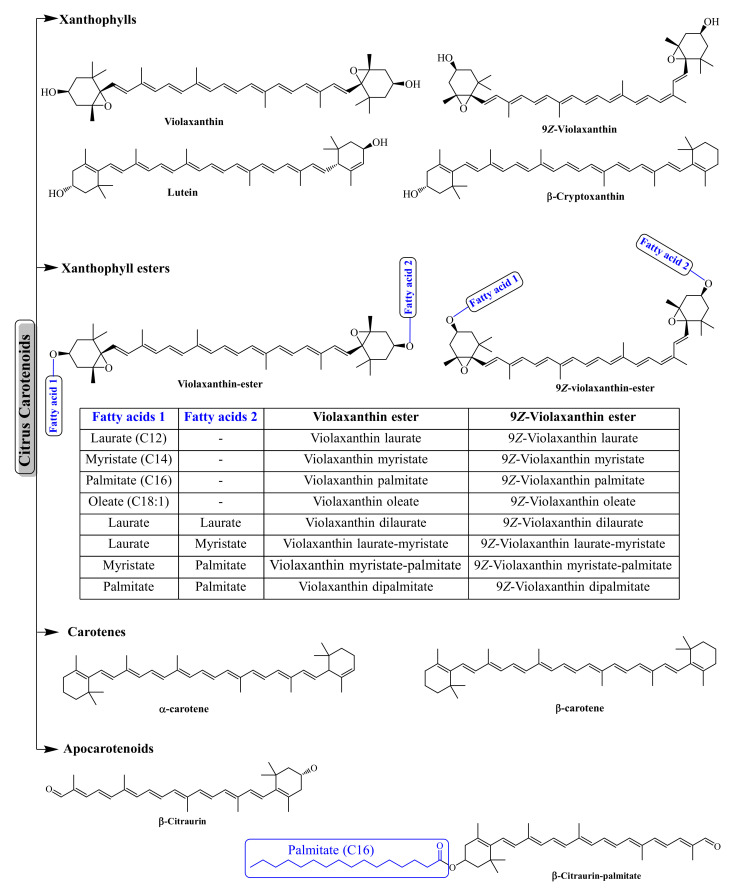
The major carotenoids and apocarotenoids of citrus fruits.

Carotenoids are a ubiquitous class of isoprenoid pigments involved in photosynthesis and signaling [42,43]. Based on their chemical structure, the carotenoids are divided into two major groups: (a) carotenes—the hydrocarbon carotenoids, such as α- and β-carotene, and lycopene; (b) xanthophylls—oxygenated derivatives of hydrocarbon carotenoids, such as neoxanthin, violaxanthin, lutein, and β-cryptoxanthin [42,43]. The oxygenated functional groups of xanthophylls can be esterified with fatty acids, and thus found in free or fatty acid esterified forms, while, due to the simple hydrocarbon structure, carotenes are found only in free form (no esterification possible due to the absence of oxygenated functions groups). In citrus fruits, xanthophylls are commonly acylated with saturated and unsaturated fatty acids, including caprate (C10:0), laurate (C12:0), myristate (C14:0), palmitate (C16:0), stearate (C18:0), palmitoleate (C16:1), and oleate (C18:1) acyl moieties (Figure 2) [44,45].

Apart from the carotenes and xanthophylls, apocarotenoids are another category of carotenoids. The carotenoid cleavage dioxygenases, i.e., the (CCDs)/9-cis-epoxycarotenoid dioxygenase (NCED)-mediated cleavage of carotenoids, gives rise to ecologically and nutritionally important apocarotenoids [46]. In citrus, β-citraurin is generated from the CCD4b1/CitCCD4-catalyzed asymmetric cleavage (at either position 7, 8 or 7′, 8′) of β-cryptoxanthin or zeaxanthin [47].

The presence of carotenoids and apocarotenoids confers the orange-red color to the peel and pulp of citrus fruits [47]. The carotenoid composition of citrus fruits is dominated by carotenoid fatty acid esters (xanthophyll esters) [44,45]. The occurrence of specific xanthophyll esters and total carotenoids largely depends on the species, maturity stage, and fruit parts [44,45]. For instance, at the fully mature stage, the total carotenoid contents of the flavedo of sweet orange were nine-fold higher (12.6 mg/100 g FW) than those in the pulp (1.4 mg/100 g FW) [45]. In this study, the most abundant carotenoids in the endocarp and flavedo of fully mature oranges were (all-*E*)- and (9*Z*)-violaxanthin, monoesters, and diesters esters carrying caprate, laurate, myristate, palmitate, stearate, palmitoleate, and oleate acyl moieties. The other major carotenoids were (all-*E*)-antheraxanthin, (all-*E*)-lutein, and (all-*E*)-β-carotene. In contrast, in this study, (all-*E*)-violaxanthin, (all-*E*)-lutein, (all-*E*)-α-carotene, and (all-*E*)-β-carotene were also found to be prevalent in the flavedo of fully mature green fruits. Moreover, the esters of β-citraurin were also detected in the flavedo of fully mature oranges.

In the Valencia orange fruit pulp, esters of violaxanthin, antheraxanthin, β-cryptoxanthin, and mutatoxanthin esterified mainly with laurate, myristate, and palmitate as monoesters or diesters are the most dominant carotenoids. Meanwhile, free xanthophylls and carotenes, such as α-, β-, and ζ-carotene, are found in a small amount [44]. In the extracted juice, the natural acidity of the juice catalyzes the isomerization and rearrangement of 5,6-epoxy-carotenoids (e.g., violaxanthin and antheraxanthin) to their respective 5,8-epoxy-carotenoids (e.g., luteoxanthin, mutatoxanthin, auroxanthin) [44]. Although xanthophylls dominate in the citrus fruits, a substantial amount of lycopene is also found in the red-fleshed pomelo and grapefruit [48,49].

The β-cryptoxanthin content is commonly used to distinguish mandarins from sweet oranges [50]. In sweet orange (*cv*. ‘Pêra’), (9*Z*)-violaxanthin, and (9′*Z*)- or (9*Z*)-antheraxanthin dominate, while it is low in (all-*E*)-β-cryptoxanthin [50]. In contrast, (all-*E*)-β-cryptoxanthin dominates in tangor (*C. reticulata* × *C. sinensis cv*. Murcott), followed by (9*Z*)-violaxanthin. Interestingly, in this study, the hybrids between *cv*. ‘Pêra’ (female genitor) and *cv*. ‘Murcott’ (female genitor) produced several orange-like groups with low β-cryptoxanthin content, and the mandarin-like group contained the highest level of β-cryptoxanthin xanthophyll (80.6–124.8 μg/g, 25-fold higher than sweet orange and twice that of tangor), suggesting the transgressive segregation of carotenoid biosynthesis.

Similar to the phenolic compounds, the citrus fruit peel flavedo contains a higher amount of carotenoids than the juice sacs [41]. However, unlike phenolic compounds, carotenoid contents increase during maturation [41,50]. In addition, in contrast to phenolic compounds, the albedo contains only a trace amount of carotenoids [41]. In a comparative study among oranges (*cv*. Washington navel and *cv*. Tarocco), lemon (*cv*. Akragas), and pummelo (*cv*. Chandler), the highest amount of total carotenoids was recorded in the flavedo of ripened Washington navel orange (159 mg/kg DW), while, among the juice sacs of ripened fruits, the highest content of total carotenoids was recorded from Tarocco orange (63.7 mg/kg DW). In this study, lutein was the most dominating carotenoid in the juice sacs of the studied fruits, accounting for 83% of the total carotenoids in the juice sacs of Tarocco orange, whereas violaxanthin, antheraxanthin, β-cryptoxanthin, and β-carotene were minor carotenoids.

### 3.3. Essential Oil (Terpenes and Limonoids)

The essential oil obtained mainly from the flavedo of citrus fruits is an economically important product with beneficial health activities due to the presence of terpenes and limonoids with other bioactive components, including flavonoids, carotenoids, and coumarins [51,52]. The citrus essential oils are widely used in the pharmaceutical, cosmetics, perfumery, and food industries due to their natural fruity perfumes [11,53]. Moreover, citrus essential oils possess potent antioxidant, analgesic, anxiolytic, neuroprotective, and antimicrobial activities [11,54,55]. In particular, bioactive compounds from citrus essential oil are well known for their potential antimicrobial properties, as they cause significant lysis of the bacterial cell wall, intracellular ingredient leakage, and, subsequently, cell death [54]. Due to its potent antimicrobial activities, in recent years, the citrus essential oil has received significant attention as a preservation agent of fruits, vegetables, meat, and processed food products [53].

**Figure 3:**
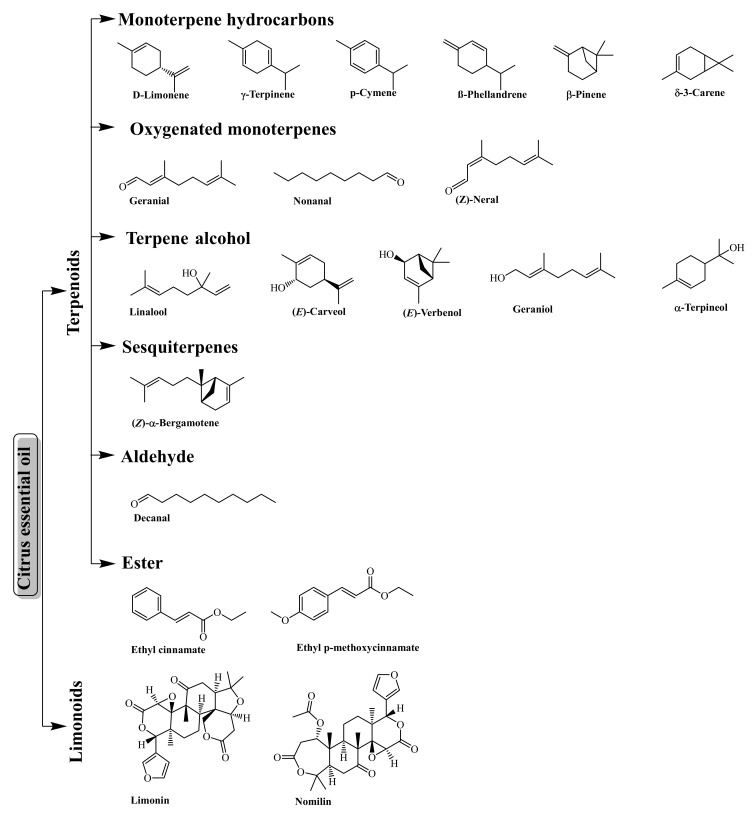
The major bioactive compounds (terpenes and limonoids) of citrus essential oil.

The monoterpene hydrocarbons (e.g., D-limonene, γ-terpinene, p-cymene, β-phellandrene, β-pinene, δ-3-carene, myrcene), oxygenated monoterpenes (e.g., geranial, nonanal, and (*Z*)-neral), terpene alcohol (e.g., linalool, (*E*)-carveol, (*E*)-verbenol, geraniol, and α-terpineol), sesquiterpenes (e.g., (*Z*)-α-bergamotene), aldehyde (e.g., decanal), and esters (e.g., ethyl cinnamate and ethyl p-methoxycinnamate) are the major chemical constituents of the volatile fractions of citrus essential oil (Figure 3) [5,54]. Moreover, the non-volatile fraction (1 to 15% of cold-pressed citrus essential oil) is mainly composed of fatty acids, long-chain hydrocarbons, sterols, wax, and limonoids (e.g., limonin) [51].

In mandarin/tangerine, grapefruit, orange, citron, and lemon essential oil, D-limonene accounts for nearly 45–90% of the total terpenoids [51,54,56,57]. In lemon and mandarin essential oil, γ-terpinene and β-pinene account for 8–20% and 0.3–11% of the total compounds, respectively [51]. Among the cold-pressed citrus essential oil from lemon, bergamot, sweet orange, clementine, bitter orange, blood orange, mandarin (green, yellow, red), and pink grapefruit, the highest amount of limonin (21.2 mg/L) was recorded from bergamot essential oil, while essential oils from clementine and blood orange presented the lowest (0.5–0.9 mg/L) limonin content [58]. In this study, among the green, yellow, and red mandarin, green mandarin showed four-times higher limonin (4.5 mg/L) than yellow and red mandarin (1.1 mg/L).

In the essential oil obtained from the fruit peel of Montenegrin mandarin, D-limonene and γ-terpinene were the major fractions, with the minor presence of citronellol and β-linalool [59]. Surprisingly, in this study, the presence of these minor components favored the antioxidant activity, while colorectal cancer HT-29 cells’ cytotoxicity was significantly decreased. In a comparative study among essential oils obtained from grapefruit, lemon, mandarin, and orange, the highest 2,2′-Azino-bis(3-ethylbenzothiazoline-6-sulfonic acid (ABTS) radical cation reducing activities and ferric reducing antioxidant power (FRAP) was obtained from mandarin essential oil, while lemon essential oil showed the highest 2,2-diphenyl-1-picrylhydrazyl (DPPH) free radical scavenging and cupric ion reducing antioxidant capacity (CUPRAC) [51].

## 4. Bioactive Compounds of Citrus Fruit Byproducts

The domestic and industrial processing of citrus fruit generates a considerable amount of peel, pulp, and seeds as byproducts, called pomace. A significant amount of research has been conducted to recover the commercially vital compounds from citrus fruit pomace [60,61,62]. Citrus peel is a rich source of essential oils [63], carotenoids [64,65,66], pectin [67,68], flavonoids [69,70,71], and several other bioactive components with excellent antioxidant [69] and health-promoting potential [62,72,73,74]. Among the flavonoids, hesperidin, naringin, rutin, and neohesperidin are the major flavonoids found in the peel of citrus fruits [71,74], with especially high amounts in mandarins, which exhibit high antioxidant potency [71]. Surprisingly, the peel of most citrus fruits contains more polyphenols and other antioxidant compounds than edible pulp [65,75]. Therefore, peels from citrus fruits can potentially be used to recover these health-beneficial compounds. Moreover, given the low lignin content, the citrus peel can serve as a promising alternative to lignocellulosic biomass to produce biofuels [76].

Similar to citrus peel, the seeds are rich in nutritionally vital proteins [73], ascorbic acid [65], fatty acids, phytosterols and tocopherols, limonoids, dietary fibers, and flavonoids [65,77]. A comparative study among the seeds, peel, and pulp of fruits of three cultivars of mandarins, including Phlegraean mandarin (*C. reticulata*), kumquat (*C. japonica*), and clementine (*C clementina*), revealed that Phlegraean mandarin is richer in bioactive compounds, including total polyphenols (seeds > peel > pulp), antioxidant activity (seeds > peel > pulp), and ascorbic acid (seeds > peel > pulp) [65].

Hesperidin and narirutin are the major flavonoids of *C. unshiu* peel [78,79]. Using the response surface model (RSM), the optimal extraction temperatures for the semi-continuous subcritical water extraction (SWE) of hesperidin and narirutin from *C. unshiu* peel were predicted as 164.4 °C and 154.6 °C, respectively, with an optimal flow rate of 2.25 mL/min. With these extraction conditions, the predicted yields of hesperidin and narirutin were 45.2 and 8.76 mg/g DW, respectively, corresponding to a recovery rate of 90.4% and 94.4%, respectively. In another study, Hwang et al. [78] optimized the extraction of hesperidin and narirutin from *C. unshiu* peel using SWE aided by pulsed electric field (PEF) treatments. In this study, PEF treatment for 2 min, combined with SWE at 150 °C for 15 min, provided the highest (46.96 mg/g DW) yield of hesperidin, while the narirutin yield was highest (8.76 mg/g DW) after PEF treatment for 2 min, combined with SWE at 190 °C for 5 min.

In view of the above, citrus pomace presents enormous opportunities to recover bioactive compounds and has a wide range of commercial applications in the food, feed, and pharmaceuticals industries. Moreover, the utilization of citrus pomace can create a surplus revenue that can substantially improve the economics of citrus fruit processing.

## 5. Health Benefits of Citrus Fruit Bioactive Compounds

### 5.1. In Vitro Studies

#### 5.1.1. Flavonoids

The antioxidant activity of citrus bioactive compounds, especially flavonoids, carotenoids, terpenes, and limonoids, can attenuate oxidative stress-related disorders [80,81]; they thus have potential applications against obesity [82], inflammatory diseases [32,83,84], atherosclerosis [85], neurodegenerative diseases [86,87,88], and cancer [89,90] (Table 3).

The pancreatic lipase (PL) is a crucial enzyme involved in triglycerides’ hydrolysis in the gastrointestinal tract, and its inhibition can ameliorate obesity by minimizing lipid absorption [82]. Hesperidin, neohesperidin, naringin, narirutin, and eriocitrin were found to be the major components in the citrus peel extracts of grapefruit, pomelo, kumquat, mandarin, and ponkan [82]. Interestingly, in this study, among these flavonoids, hesperidin, the most dominant flavonoid in ponkan peel extract, showed the highest pancreatic lipase inhibition activities, suggesting its promising application in managing obesity.

Citrus peel flavonoid nobiletin suppresses the inflammatory response in lipopolysaccharide (LPS)-stimulated RAW264.7 cells by enhancing autophagy through decreasing the levels of inflammatory cytokines (inducible nitric oxide synthase (iNOS) and cyclooxygenase-2 (COX-2)) and activating the Interleukin-6 (IL-6)/signal transducer and activator of transcription 3 (STAT3)/forkhead box O3a (FOXO3a) signal pathway responsible for the induction of macrophage autophagy [32].

Dysregulation of IL-5 secretion by antigen-specific T helper 2 (Th2) cells has been linked to eosinophilic inflammation in asthma [84]. The Th2 cytokine expression is regulated by transcription factors, including the nuclear factor of activated T cells (NFAT) [84]. Moreover, Heme oxygenase-1 (HO-1) expression is known to suppress the asthmatic immune response modulation of phosphoinositide 3-kinase (PI3K)/protein kinase B (AKT), extracellular signal-regulated kinases (ERK)/c-Jun N-terminal kinases (JNK), nuclear factor erythroid 2 related factor 2 (Nrf2), and peroxisome proliferator-activated receptor γ (PPARγ) signaling [84]. Citrus flavonoid gardenin A and hesperidin exhibited a robust suppressive effect on IL-5 secretion by PMA/ionomycin-treated EL-4 murine T-lymphoma cells by downregulating NFAT protein expression [84]. In this study, hesperidin and gardenin A induced HO-1 protein expression and repressed IL-5 production through distinct pathways; hesperidin upregulated HO-1 production via Nrf2 protein expression combined with the activation of the ERK/JNK and PI3K/Akt signaling pathways, whereas gardenin A induced HO-1 expression through the transcription factor PPARγ.

Beyond the health-beneficial effects described above, bioactives present in citrus fruit juices were shown to have direct antiviral activity. Dong et al. [91] mentioned that hesperidin restricted the replication and progression of the Influenza virus in human lung carcinoma A549 cells by upregulating the p38 signaling pathway. Hesperidin, hesperetin, and naringenin were shown to inhibit key proteases involved in coronavirus replication and prevent virus entry into host cells [92,93,94].

The flavonoids (hesperidin, naringin, tangeritin, and rutin) rich in the hydro-ethanolic extract of *C. reticulata* Blanco peels have shown antiproliferative effects against BT-474 human breast carcinoma [95]. In this study, 500 μg/mL of extract treatment reduced the viability of BT-474 cells by 47% and 60% after 24 or 48 h of treatment, respectively.

#### 5.1.2. Carotenoids

Carotenoids are widely investigated for their anticancer activities [96,97]. Carotenoids are well known for their antioxidant function in the normal cellular environment [42]. However, in cancer cells with an innately high intracellular ROS level, carotenoids may act as potent pro-oxidant molecules and promote ROS-mediated apoptosis [98]. In our study, we have demonstrated that the anticancer activities of β-cryptoxanthin derived from mandarin oranges on human cervical carcinoma (HeLa) cells are mediated through pro-oxidant action, which enhances the ROS generation, followed by the enhanced expression of caspase-3, -7, and -9, Bax, and p-53 at the mRNA, with the concordant suppression of antiapoptotic Bcl-2. These events trigger the nuclear condensation, loss of mitochondrial membrane potential, activation of caspase-3 proteins, and, finally, cleavage of nuclei DNA. In this study, β-cryptoxanthin substantially inhibited the proliferation of HeLa cells, with an IC50 value of 4.5 µM after 24 h of treatment.

#### 5.1.3. Essential Oil (Terpenes and Limonoids)

The lumy essential oil rich in limonene (48.9%) and linalool (18.2%) has been shown to exhibit potent antioxidant and free radical scavenging properties with anti-acetylcholinesterase activities [86]. Moreover, in this study, lumy essential oil showed neuroactive effects by significantly reducing the burst frequency (MBR), assessed by the spontaneous electrical activity of rat cortical neuronal networks.

In the neuronal cells, K^+^ imbalance, activation (phosphorylation) of extracellular signal-regulated protein kinase (ERK1), and reactive oxygen species (ROS) production are associated with the progression of Alzheimer’s disease (AD) [87]. In addition, the acetylcholinesterase (AChE) enzyme involved in the hydrolysis of acetylcholine plays a vital role in triggering neuropsychiatric symptoms in AD [87]. Limonene has shown protective effects in Aβ_1–42_ oligomer-triggered toxicity in primary cortical neurons (in vitro model of AD) by suppressing the AChE, ROS production, and voltage-gated K^+^ channel KV3.4 hyperfunction, and downregulating phosphorylated (p)-ERK [87].

Citrus limonoids (limonexic acid, limonin, and nomilin) have been shown to induce apoptosis and inhibit the proliferation (IC50 values < 50 μM after 72 h) of pancreatic cancer cells Panc-28, by the enhanced cleavage of caspase-3, decreased mitochondrial membrane potential, and upregulation of the expression of B-cell lymphoma 2 (Bcl-2)-associated X protein (Bax)/Bcl-2 proteins [90]. Moreover, in this study, limonoids upregulated the expression of cyclin-dependent kinase inhibitor (p21) and exhibited anti-inflammatory activity through downregulating the expression of proinflammatory proteins Cox-2, nuclear factor-kappa β (NF-κB), and IL-6.

#### 5.1.4. Other Bioactives

Apart from the flavonoids, carotenoids, terpenes, and limonoids, citrus pectin and coumarin have beneficial health properties [85,99]. For instance, citrus pectin oligosaccharides and their microbial metabolites exhibited anti-atherosclerosis effects on LPS-treated human macrophages by regulating the expression of proinflammatory mediators (TNF-α, IL-6, IL-10, and NF-κB mRNA) [85]. Moreover, in this study, cholesterol efflux was also accelerated by the upregulation of the liver X receptor-α (LXRα) and adenosine triphosphate-binding cassette transporter (ABC) A1 and G1 (ABCG1) mRNA. Furthermore, pectin oligosaccharides repressed cholesterol synthesis by the downregulation of 3-hydroxy-3-methylglutaryl-coenzyme A reductase (HMGCR) mRNA.

The coumarins isolated from pomelo have shown hepatoprotective activities in D-galactosamine-treated normal human hepatic LO2 cells by suppressing the levels of alanine transaminase (ALT) and aspartate transaminase (AST), increasing the activities of antioxidant enzymes, including glutathione peroxidase (GSH-Px) and superoxide dismutase (SOD), and decreasing the level of malondialdehyde (MDA) [99].

**Table 3:** Health benefits of citrus fruit bioactive compounds demonstrated using in vitro experimental models.

Compounds	Experimental System	Disease Target	Mechanism of Action	Reference
Flavanone-rich mandarin juice extract (0.001–1 mg/mL)	6-hydroxydopamine (6-OHDA)-stimulated SH-SY5Y human neuroblastoma cells	Parkinson’s disease (PD)	↓ROS and NO, restored SOD and CAT activity, ↓caspase 3 activity, ↑Bcl-2 mRNA, ↓p53 and Bax mRNA, restored mitochondrial membrane potential, ↓oxidative DNA damage, balanced α-synuclein, LRRK2, parkin, PINK1, and DJ-1 mRNA levels	[88]
Flavanones (10 µM)	Caco-2 cells stimulated with IL-1	Bowel diseases	↓IL-6, IL-8, and NO release	[83]
Hesperetin and gardenin A (5–10 µM)	PMA/ ionomycin-induced EL-4 murine T-lymphoma cell cells	Asthma	↓ROS and IL-5 production, ↓NFAT activity and IL-5 secretion, ↑HO-1 through ↑Nrf2, PPARγ, PI3K/AKT, or ERK/JNK signaling	[84]
Limonene (1–100 mg/mL)	Aβ_1–42_ triggered toxicity in primary cortical neurons	Alzheimer’s disease (AD)	↓AchE, ROS production, voltage-gated K+ channel KV3.4 hyperfunction, and phosphorylated ERK	[87]
Limonin, nomilin, and limonexic acid (20–60 µM)	Human pancreatic Panc-28 cells	Cancer (pancreatic)	↓Cell proliferation (IC_50_ values < 50 μm after 72 h), ↑cleavage of caspase-3, mitochondrial membrane potential, ↑Bax/Bcl2 expression, and p21, ↓COX-2, NF-κβ, and IL-6	[90]
Limonoids (Fortunellon and nomilin; 30 µM)	HeLa cells	Cancer (Cervical)	↑Adriamycin-dependent cell death	[89]
Naringenin (62.5–2000 µM)	Human A549 lung epithelial cells and primary human monocyte-derived dendritic cells	Zika virus infection	↓Replication or assembly of viral particles	[100]
Naringin- and hesperidin-rich junos peel extract (0.5 mg/mL)	Human lung basal epithelial NCI-H460 cells exposed to H_2_O_2_	Oxidative stress-induced diseases	↓p53, cytochrome c, and Bax proteins	[80]
Pectin oligosaccharides (5 mg/mL)	LPS-stimulated human macrophages	Atherosclerosis	↑Immune responses, ↓TNF-α, IL-6, IL-10, and NF-κβ mRNA, ↑cholesterol efflux via LXRα and ABCA1, and ABCG1 pathway, ↓cholesterol synthesis via ↓HMGCR	[85]
Phase-II flavanone metabolites (2–100 µM)	Pancreatic β-cell MIN6 cells exposed to cholesterol	Oxidative stress-induced diseases	↓Oxidative biomarkers (superoxide anion, H_2_O_2_, and MDA), ↓SOD and GPx, ↑insulin secretion, ↓apoptosis	[81]
PMF nobiletin (10–50 µM)	LPS-stimulated RAW264.7 cells	Inflammatory diseases	↓Release of NO, ↓expression of iNOS and COX-2, ↑autophagy, activation of the IL-6/STAT3/FOXO3a signal pathway	[32]
β-cryptoxanthin from mandarin oranges; IC_50—_4.5µM (24 h treatment)	HeLa cells	Cancer (cervical)	↓Bcl-2 mRNA, ↑Bax, caspase-3, -7, and -9 mRNA, nuclear condensation and disruption of the integrity of the mitochondrial membrane, activation of caspase-3 proteins, nuclei DNA damage, and apoptosis	[101]

The upregulation and downregulation are denoted by upward (↑) and downward (↓) arrows, respectively. Abbreviations are as follows: ABCA1: adenosine triphosphate-binding cassette transporter subfamily A member 1; ABCG1: adenosine triphosphate-binding cassette transporter subfamily G member 1; AChE: acetylcholinesterase; AKT: protein kinase B; Aβ_1–42_: amyloid β-protein; Bax: B-cell lymphoma 2 (Bcl-2)-associated X protein; Bcl2: B-cell lymphoma 2; CAT: catalase; COX-2: cyclooxygenase-2; DJ: Parkinson disease protein 1; EPO: eosinophil peroxidase; ERK: extracellular signal-regulated kinases; FOXO3a: Forkhead box O3a; GPx: glutathione peroxidase; H_2_O_2_: hydrogen peroxide; HeLa: human cervical cancer cells; HMGCR: 3-hydroxy-3-methylglutaryl-coenzyme A reductase; HO-1: Heme oxygenase-1; IL: Interleukin; iNOS: inducible nitric oxide synthase; JNK: c-Jun N-terminal kinases; LPS: lipopolysaccharide; LRRK2: leucine-rich repeat kinase 2; LXRα: liver X receptor-α; MDA: malondialdehyde; MPO: myeloperoxidase; NFAT: nuclear factor of activated T cells; NF-κβ: nuclear factor-kappa β; NO: nitric oxide; Nrf2: nuclear factor-erythroid 2 related factor 2; PI3K: phosphoinositide 3-kinase; PINK1: phosphatase and tensin homolog (PTEN)-induced putative kinase 1; PMA: phorbol 12-myristate 13-acetate; PMF: polymethoxylated flavone; PPARγ: peroxisome proliferator-activated receptor γ; ROS: reactive oxygen species; SOD: superoxide dismutase; STAT3: signal transducer and activator of transcription 3; TNF-α: tumor necrosis factor α.

### 5.2. In Vivo Studies

Excess caloric supply causes chronic hyperlipidemia and hyperglycemia, triggering atherosclerosis, hepatic steatosis, obesity, diabetes, and cardiovascular complications [102,103,104]. These metabolic diseases are linked to a wide array of metabolic complications [102]. Hyperlipidemia is the condition of disorder of lipid metabolism, resulting in abnormally elevated levels of low-density lipoprotein cholesterol (LDL-c) and very-low-density lipoprotein cholesterol (VLDL-c), triglyceride (TG), and total cholesterol (TC) in the blood, as well as reduced levels of high-density lipoprotein cholesterol (HDL-c) [102]. Similarly, chronic hyperglycemia is the condition of persistent and unusually high postprandial (after a meal) blood glucose levels, primarily due to the flawed insulin production [104]. Several recent animal studies have demonstrated the beneficial effects of citrus flavonoids, carotenoids, terpenes, limonoids, and other bioactives (e.g., pectin and coumarins) against metabolic syndrome (Table 4). Moreover, the potent antioxidant activities of citrus bioactives have shown protection against primary dysmenorrhea (PD) [105], pulmonary edema [80], cancer [36], and neuropsychiatric [106] and neurodegenerative diseases [107,108].

#### 5.2.1. Flavonoids

The citrus flavonoids are most widely investigated for their antihyperglycemic and antihyperlipidemic effects in animal models [104,108]. Citrus flavonoids, such as hesperetin, have shown potential in attenuating hyperglycemia in streptozotocin (STZ)-induced diabetes in rats by releasing insulin from β cells of islets [104]. In this study, hesperetin supplementation of 40 mg/kg for 45 days showed a significant decrease in plasma glucose levels and a significant increase in the level of plasma insulin. It restored the compromised antioxidant status by increasing the activity of SOD, catalase (CAT), and glutathione peroxidase (GPx). Moreover, in this study, hesperetin alleviated hyperlipidemia by lowering the cholesterol, free fatty acid (FFA), TG, and phospholipid (PL) levels in diabetic rats, probably via the insulin-mediated reduction in the synthesis of fatty acids and cholesterol. Moreover, the authors suggested that the cholesterol-lowering effect of hesperetin is possibly due to the capability of hesperetin and other flavonoids to bind to bile acids, resulting in enhanced bile acid secretion and a reduction in cholesterol absorption [104].

The flavanone aglycones present in fermented/non-fermented ougan (*cv*. Suavissima) juice exhibited anti-obesity properties in high-fat-diet (HFD)-fed C57BL/6J mice by reduced weight gain, decreased fat accumulation, enhanced glucose homeostasis and insulin sensitivity, lowered liver steatosis, enhanced white adipose tissue (WAT) browning, augmented brown adipose tissue (BAT) activity, and increased diversity of gut microbiota [109].

The gastrointestinal microbiota composition plays a vital role in host physiology, nutrition, and metabolism [110]. Changes in the gastrointestinal microbiota composition, the community of pathogenic symbiotic and microorganisms, are probably responsible for the anti-obesity effects of citrus bioactives, especially flavonoids [35,111]. The abundance of gut microbiota, *Firmicutes* over *Bacteroidetes*, is linked to obesity-related metabolic syndrome [35]. Moreover, the gut microbiome’s branched-chain amino acid (BCAA) metabolism is considered responsible for metabolic syndrome [35]. It is likely that microbially produced BCAAs, such as imidazole propionate, impair insulin signaling through the activation of mammalian target of rapamycin (mTOR) complex 1 (mTORC1) and P70S6K [35]. Sterol regulatory element-binding proteins (SREBPs) play essential roles in regulating lipid homeostasis via mTOR. An extract rich in PMFs and hydroxy polymethoxyflavones (HOPMFs) (0.5% of HFD for 16 weeks) from citrus peel attenuated the obesity and modulated gut microbiota in male C57BL/6 mice fed a HFD by altering the gut microbiota, by increasing *Prevotella* and decreasing rc4–4 bacteria [111]. In this study, PMFs and HOPMFs alleviated the total body weight, decreased the lipids in 3T3-L1 preadipocytes, and reduced the adipocyte size and adipose tissue weight in the HFD mice. Moreover, in this study, PMFs and HOPMFs decreased the levels of lipid droplets (LD) and perilipin 1 protein and sterol regulatory element-binding protein 1 (SREBP-1) expression. Similarly, in another study, a citrus PMF (nobiletin and tangeretin)-rich extract was shown to ameliorate HFD-induced metabolic syndrome via gut dysbiosis (decreased *Firmicutes*-to-*Bacteroidetes* ratio), and regulated branched-chain amino acid (BCAA) metabolism [35]. In this study, the PMF-rich extract inhibited the phosphorylation of mTOR and P70S6K and decreased the expression of SREBPs in human liver HL-7702 cells and HFD-fed mice. Therefore, the authors hypothesized that the decreased BCAAs by the PMF-rich extract contribute to improving metabolic syndrome by inhibiting the mTOR/P70S6K/SREBP pathway.

Among the four flavanones tested for their anti-atherosclerosis potential in apolipoprotein E-deficient (ApoE^−/−^) mice, naringin showed the most potent anti-atherogenic effect, followed by hesperidin, naringenin, and hesperetin [33]. In this study, oral naringin admiration alleviated atherosclerosis by enhancing bile acid synthesis. Hesperidin upregulated ABCA1 to enhance cholesterol reverse transport, while the aglycones naringenin and hesperetin inhibited cholesterol synthesis significantly by downregulating 3-hydroxy-3-methyl-glutaryl-coenzyme A reductase (HMGCR).

Dietary administration of 0.05% PMF 5-demethylnobiletin has shown chemopreventive effects against azoxymethane/dextran sulfate sodium (DSS)-driven colorectal carcinogenesis in male CD-1 mice by reduced cell proliferation, increased apoptosis, and decreased mRNA and protein levels of proinflammatory cytokines IL-1β, IL-6, and TNF-α in the colon [36]. In this study, a significant amount of 5-Demethylnobiletin metabolites, namely 5,3′-didemethylnobiletin, 5,4′-didemethylnobiletin, and 5,3′,4′-tridemethylnobiletin, was documented in the colonic mucosa of the treated mice. Surprisingly, these metabolites showed more potent effects than 5-demethylnobiletin on inhibiting the proliferation, inducing cell cycle arrest, and the apoptosis of HCT116 human colorectal cancer cells.

Higher levels of circulating thyroid-stimulating hormone (TSH) are vital for greater longevity [112]. The upregulation of sirtuin 1, which deacetylates transcription factors that contribute to cellular regulation, may positively upregulate the exocytosis of TSH-containing granules [112]. Due to the antioxidant and anti-inflammatory properties, 15 mg/kg body mass (BM) of citrus naringenin has shown increased TSH secretion in 24-month-old male Wistar rats by upregulating the Sirt1 protein expression [112].

Neuroinflammation is also crucial in several neurodegenerative disorders, including Alzheimer’s disease (AD) and Parkinson’s disease (PD). Due to its antioxidant and anti-inflammatory properties, hesperetin has shown protective effects against LPS-induced neuroinflammation, neuronal apoptosis, oxidative stress, and memory impairments in C57BL/6 N mice via regulating the toll-like receptor 4 (TLR4)/NF-κB signaling pathways [107]. In this study, hesperetin repressed the inflammatory mediators (p-NF-κβ, TNF-α, and IL-1) and ROS/lipid peroxidation, and improved the antioxidant protein levels (Nrf2 and HO-1). Moreover, hesperetin reduced neuronal apoptosis by reducing the expression of Bax, phosphorylated-c-Jun *N*-terminal kinases (p-JNK), and caspase-3 protein and upregulating the Bcl-2 protein level. Moreover, hesperetin prompted the cognition, synaptic integrity, and memory processes by augmenting the postsynaptic density protein-95 (PSD-95), phosphorylated-cAMP response element-binding protein (p-CREB), and Syntaxin.

Hyperglycemia is considered a vital risk factor in developing neurodegenerative disorders, as it is known to promote brain astroglial activation, oxidative stress, inflammation, amyloid-β-accumulation, tau hyperphosphorylation, and memory impairment [108]. Tau hyperphosphorylation induces microtubule dysfunction, leading to the formation of neurofibrillary tangles (NFTs), which are often observed in AD [108]. The citrus auraptene and naringin have shown inhibitory effects against tau hyperphosphorylation, astroglial activation, and recovered the suppression of neurogenesis in the hippocampus of STZ-induced hyperglycemic mice [108].

Chronic inflammation is involved in the etiology of several intestinal disorders, including inflammatory bowel diseases (IBDs), which mainly comprise ulcerative colitis and Crohn’s disease [113]. The *C. kawachiensis* peel powder rich in flavonoids (naringin, narirutin, and auraptene) and dietary fiber protected from the DSS-induced intestinal inflammation in a murine model of colitis [113]. In this study, supplementation of peel powder (5% of diet, *w*/*w*) ameliorated the DSS-induced body weight loss, colon shortening, increased expression of pro-inflammatory cytokines (e.g., TNF-α), and decreased expression of colonic tight junctions (TJs) (e.g., occluding).

#### 5.2.2. Carotenoids

The provitamin A carotenoids (e.g., β-cryptoxanthin) from citrus fruits have also shown effectiveness against metabolic syndromes, such as type 2 diabetes [103]. In the body (intestine and liver), provitamin A carotenoids are bio-converted to retinol by the activities of β-carotene 15,15′-oxygenase (BCO1). In a high-fructose-diet-induced type 2 diabetes model of Wistar male rats, feeding of citrus concentrate containing 0.086 mg β-cryptoxanthin, 5.69 mg hesperidin, and 7.5 mg pectin for eight weeks decreased insulinemia, glycemia, and dyslipidemia by restoring the LDL-c and TG levels to be similar to the healthy group [103]. Moreover, in this study, feeding purified β-cryptoxanthin alone or with a matrix containing hesperidin and pectin showed the synergy between these constituents. Furthermore, in this study, β-cryptoxanthin from citrus fruits was shown to restore the vitamin A status in both control and prediabetic (high-fructose fed) rats; however, prediabetic rats showed lower absorption bioconversion of β-cryptoxanthin into retinoids.

The synergy between carotenoids and flavonoids is probably due to the enhanced uptake of carotenoids in the presence of flavonoid glycosides [114]. In Caco-2 cells, it has been shown that flavanone O-glycosylation (at C_7_ of the A ring) led to the highest promoting effect on β-carotene absorption via enhanced paracellular permeability by transient drop-in tight junction (TJ) protein expression, and the upregulation of peroxisome proliferator-activated receptor-gamma (PPARγ) and scavenger receptor class B type I (SR-BI; proteins involved in carotenoid absorption and transport) expression [114].

#### 5.2.3. Essential Oil (Terpenes and Limonoids)

The overproduction of endometrial prostaglandins (PGs), especially prostaglandin F2α (PGF2α) and prostaglandin E2 (PGE2), is considered to be one of the critical factors for the progression of primary dysmenorrhea (PD) [105]. A higher ratio of PGF2α/PGE2 is considered to be a principal indicator of PD [105]. The citrus essential oil, particularly sweet orange essential oil rich in limonene, exhibited relief from estradiol benzoate- and oxytocin-induced PD in female Sprague Dawley rats via decreasing the level of PGF2α and increasing PGE2, resulting in a decrease in the ratio of PGF2α/PGE2. Moreover, in this study, citrus essential oil prevented a decrease in antioxidant status markers, including total antioxidant capacity (T-AOC), SOD, and CAT, and an increase in MDA levels.

Anxiety and depression are the most common forms of neuropsychiatric disorders [106]. The essential oil from oranges and its main component limonene have shown an antidepressant-like effect in a chronic unpredictable mild stress (CUMS) male Kunming mice mouse model by restoring the decreased curiosity and mobility, reduced body weight gain, reduced sucrose preference, decreased levels of monoamine neurotransmitter 5-hydroxytryptamine (5-HT), dopamine (DA), norepinephrine (NE), and brain-derived neurotrophic factor (BNDF) and its receptor tropomyosin receptor kinase B (TrkB) expression in the hippocampus, and increased levels of corticotropin-releasing factor (CRF) and corticosterone (CORT) [106].

The peel oil of mandarin, rich in limonene, myrcene, and carotenoids, has led to the dose-dependent growth inhibition of A549 non-small-cell lung cancer (NSCLC) cells and tumor growth in nude mice implemented with A479 cells [115]. In this study, supplementation of 5.25 mg/d of peel oil per mouse for seven days significantly decreased tumor growth by reducing the expression of membrane-bound Ras protein, increasing apoptosis, and inducing cell cycle arrest at the G0/G1 phase.

**Table 4:** Health benefits of citrus fruit bioactive compounds demonstrated using the animal models.

Bioactive and Doses	Experimental System	Disease Target	Mechanism of Action	Reference
Auraptene (50 mg/kg), naringin (50 mg/kg) for 14 days	STZ-induced hyperglycemia in C57BL/6 mice	Alzheimer’s disease (AD)	↓Tau hyperphosphorylation, astroglial activation, and ↑neurogenesis in the hippocampus	[108]
Citrus concentrate containing 0.086 mg β-cryptoxanthin, 5.69 mg hesperidin, and 7.5 mg pectin for 8 weeks	Wistar male rats fed with high-fructose diet	Metabolic syndrome (type 2 diabetes)	↓Plasma glucose, glycemia, insulinemia, and LDL-C, VLDL-C, and TG levels, ↑liver retinyl palmitate, and plasma β-cryptoxanthin	[103]
Coumarin (auraptene, 7.5–30 mg/kg for three days a week for total of 8 weeks)	TAA-induced hepatic fibrosis in male C57BL/6 mice	Hepatic fibrosis (cirrhosis and liver cancer)	↓Bile acids in liver by increasing their efflux, ↓activation of HSCs by suppressing the expression of TGF-β1 and -SMAα and ↓expression of NF-κB, TNF-α, and IL-1β	[116]
Coumarin auraptene (5–20 mg/kg)	17α-Ethinylestradiol (synthetic estrogen) induced cholestasis in male C57BL/6 mice	Estrogen-induced cholestasis	↑Bile acid transporters (Bsep and Mrp2) mRNA and proteins, ↑Shp and Fgf15, FXR, ↑bile acid metabolism, ↑SULT2A1, ↓Cyp7a1 and Cyp8b1 mRNA, ↓hepatic inflammation (↓TNF-α, IL-1β, and IL-6)	[117]
Essential oil (0.75% of the diet for 42 weeks)	Male SD rats fed with HFD	Metabolic syndrome (hyperlipidemia)	↓TC, LDL-C, hepatic TC, TG, and hepatic lipid droplet accumulation, ↓liver FFAs, TG, and CE	[102]
Essential oil (limonene; daily inhalation for 1.5 and 24 h, for five days)	CUMS male Kunming mice mouse model	Depression	↑Curiosity, body weight, sucrose preference, 5-HT, DA, NE, BNDF, TrkB, GR, ↓CRF, CORT	[106]
Flavanone aglycones rich ougan (*cv*. Suavissima) juice (20 mL/kg for 10 weeks)	HFD-fed C57BL/6J mice	Metabolic syndrome (obesity)	↓Weight gain, ↓fat accumulation, ↓liver steatosis, ↑glucose homeostasis and insulin sensitivity, ↑BAT activity, and ↑WAT browning, ↑diversity of gut microbiota	[109]
Flavanones (eriocitrin and eriodictyol), 25 and 50 mg/kg	BALB/c mice with LPS-induced periodontal disease	Periodontitis	↓Gingival IL-1β and TNF-α, ↑IL-10, ↓MPO and EPO activity, SOD, ↑CAT and GPx activities, ↓MDA	[118]
Flavanones (naringin, naringenin, hesperidin, and hesperetin; 100 mg/kg/day for 16 weeks)	ApoE^−/−^ mice	Atherosclerosis	↑Bile acid synthesis (naringin), ↑cholesterol reverse transport (hesperidin), ↓cholesterol synthesis (naringenin and hesperetin)	[33]
Flavonoid-rich bitter/sour orange fruit peel extract (125–500 mg/kg for 3 days)	TNBS-induced IBD in male Sprague/Dawley (SD) rats	IBD	↓Weight loss and diarrhea, colitis inflammatory cell infiltration, and proinflammatory cytokines (TNF-α, iNOS, COX-2), ↓serum and colon NO and MPO activity	[119]
Hesperetin (40 mg/kg for 45 days)	STZ-induced diabetes in male albino Wistar strain rats	Diabetes	↓Plasma glucose, ↑plasma insulin and glycogen, ↑antioxidant system (↑SOD, CAT, GPx), ↑insulin secretion by renovating pancreatic β-cells, ↓dyslipidemia (hepatic cholesterol, FFAs, TG, and PLs), ↓serum levels of ALT, AST, and ALP, ↓renal damage (serum urea, creatinine, and uric acid)	[104]
Hesperetin (50 mg/kg daily for five weeks)	LPS-induced neuroinflammation C57BL/6 N mice	Alzheimer’s disease (AD) and Parkinson’s disease (PD)	↓Inflammatory mediators (phosphorylated-NF-κβ, TNF-α, and IL-1), ROS/lipid peroxidation, ↑antioxidant protein (Nrf2 and HO-1), ↓phosphorylated-JNK, Bax, and caspase-3 protein, ↑Bcl-2, ↑synaptic integrity, cognition, and memory processes, ↑ phosphorylated-CREB, PSD-95, and Syntaxin	[107]
Hesperetin (50 mg/kg/day for 46 days)	STZ-induced diabetes in male Wistar rats	Diabetes-associated testicular injury	↓Body weight loss, ↓ serum glucose, ↓MDA, ROS, protein carbonyl, DNA fragmentation, and caspase 3 activity, ↑testicular antioxidant system (↑GSH, MMP, FRAP, SOD, CAT, GPx)	[120]
Hesperidin (100 mg/kg for eight weeks)	Male SD rats fed an obesogenic cafeteria diet	Metabolic syndrome (obesity)	↓TC, LDL-C, FFAs, MCP-1	[121]
Limonene-rich essential oil (0.0765 mL/kg for 7 days)	SD rats with estradiol benzoate and oxytocin-induced uterine contraction	Primary dysmenorrhea (PD)	↑Antioxidant status markers (SOD,T-AOC, CAT, and GSH), ↓MDA and iNOS, and PGF2α/PGE2	[105]
Naringin- and hesperidin-rich *C. junos* peel extract (200 mg/kg/day, 10 days	Acrolein-induced pulmonary apoptosis in male C57BL/6J mice	Pulmonary edema and COPD	↓Cleaved caspase 3, cleaved PARP, Bax and PUMA, p53, Prx-SO3	[80]
Pectin oligosaccharides (0.15–0.9 g/kg for 30 days)	Male C57BL/6 mice fed with HFD	Metabolic syndrome	↓Serum TC, LDL-C, ↓*Firmicutes* ↑*Bacteroidetes* ↑SCFAs (acetate, propionate, and butyrate)	[122]
PMF (nobilitin, tangeritin)-rich extract (30–120 mg/kg)	C57BL/6J male mice fed with HFD	Metabolic syndrome	↓*Firmicutes*-to-*Bacteroidetes* ratio, ↓serum BCAA, ↓mTORC1 and P70S6K activation, ↓SREBPs	[35]
PMF (nobilitin, tangeritin, and 5-OH nobiletin)-rich aged chenpi peel extract (0.25 and 0.5% of diet weight for 11 weeks)	Male C57BL/6J mice fed with HFD	Metabolic syndrome (obesity)	↑Fecal SCFAs (acetic acid and propionic acid), ↑healthy gut microbiota	[34]
PMF 5-Demethylnobiletin (12 mg per kg)	Azoxymethane/DSS-driven colorectal carcinogenesis in male CD-1 mice	Cancer (colorectal)	↓Cell proliferation, ↑apoptosis, and ↓mRNA and protein levels of IL-1β, IL-6, and TNF-α in the colon	[36]
PMF- and HOPMF-rich extract (0.5% of HFD for 16 weeks)	Male C57BL/6 mice fed with HFD	Metabolic syndrome (obesity)	↓Adipocyte size, adipose tissue weight, and alleviated the total body weight, levels of lipid droplets, and perilipin 1 protein and SREBP-1 expression, ↑gut microbiota *Prevotella*, ↓rc4–4 bacteria	[111]

The upregulation and downregulation are denoted by upward (↑) and downward (↓) arrows, respectively. Abbreviations are as follows: 5-HT: 5-hydroxytryptamine; ALP: alkaline phosphatase; ApoE: apolipoprotein E; AST: aspartate aminotransferase; BAT: brown adipose tissue; Bax: B-cell lymphoma 2 (Bcl-2)-associated X protein; BCAA: branched-chain amino acid; Bcl-2: B-cell lymphoma 2; BNDF: brain-derived neurotrophic factor; Bsep: bile salt export pump; CAT: catalase; CE: cholesterol esters; COPD: obstructive pulmonary disease; CORT: corticosterone; COX-2: cyclooxygenase-2; CREB: cAMP response element-binding protein; CRF: corticotropin-releasing factor (CRF); CUMS: chronic unpredictable mild stress; Cyp7a1: cholesterol 7α-hydroxylase; Cyp8b1: sterol-12α-hydroxylase; DA: dopamine; DSS: dextran sulfate sodium; FFA: free fatty acids; FFAs: free fatty acids; Fgf15: fibroblast growth factor 15; FRAP: ferric reducing antioxidant; FXR: farnesoid X receptor; GPx: glutathione peroxidase; GR: glucocorticoid receptor; GSH: glutathione; HFD: high-fat diet;.HO-1: Heme oxygenase; HOPMFs: hydroxy polymethoxyflavones; HSCs hepatic stellate cells; HSCs: hepatic stellate cells; IBD: inflammatory bowel disease; IL: Interleukin; iNOS: inducible nitric oxide synthase; JNK: c-Jun N-terminal kinases; LDL-C: low-density lipoprotein cholesterol (LDL-c); LPS: lipopolysaccharide; MCP-1: monocyte chemoattractant protein 1; MDA: malondialdehyde; MMP: mitochondrial membrane potential; MPO: myeloperoxidase; Mrp2: multidrug-resistance-related protein 2; mTOR: mammalian target of rapamycin; mTORC1: mammalian target of rapamycin (mTOR) complex1; NE: norepinephrine; NF-κβ: nuclear factor-kappa β; NO: nitric oxide; Nrf2: nuclear factor-erythroid 2 related factor 2; P70S6K: phospho-p70 S6 kinase; PARP: poly (ADP-ribose) polymerase; PGE2: prostaglandin E2; PGF2α: prostaglandin F2α;.PLs: phospholipids; PMF: polymethoxyflavones; Prx-SO3: oxidized peroxiredoxin; PSD-95: postsynaptic density protein-95; PUMA: p53 upregulated modulator of apoptosis; ROS: reactive oxygen species; SCFAs: short-chain fatty acids; SD: Sprague Dawley; Shp: small heterodimer partner; SMAα: α-smooth muscle actin; SOD: superoxide dismutase; SREBP-1Sterol regulatory element-binding protein 1; STZ: streptozotocin;SULT2A1: sulfotransferase family 2a member 1; TAA: thioacetamide; T-AOC: total antioxidant capacity; TC: total cholesterol; TG: triglyceride and hepatic lipid droplet accumulation; TGF-β1: transforming growth factor-β1; TNBS: trinitrobenzene sulfonic acid; TNF-α: tumor necrosis factor α; TrkB: tropomyosin receptor kinase B; VLDL-C: very-low-density lipoprotein-cholesterol; WAT: white adipose tissue.

### 5.3. Human Studies

Similar to the in vitro and animal studies, case–control, cohort, and interventional studies have also demonstrated the health benefits of bioactive compounds derived from citrus fruits. A pooled meta-analysis of 14 case–control (13 hospital-based and two population-based) and two cohort studies showed that people with the highest citrus fruit intake had a 50% reduction in risk of oral cavity and pharyngeal cancer compared to the lowest intake [123]. In this meta-analysis, the protective effect of citrus fruit was substantially higher in case–control studies (OR 0.47; 95% CI 0.40–0.55) compared to cohort studies (OR 0.73; 95% CI 0.55–0.96).

The oxidized (ox)-low-density lipoprotein (LDL) plays a vital role in converting macrophages to foam cells and the formation and progression of atherosclerotic lesions [124]. In a 3-month randomized, double-blind, controlled study, 23 untreated human subjects (16 males and seven females, mean age of 41.9 years) with cardiovascular risk (total cholesterol level >200 mg/dL and LDL-c > 130 mg/dl) consumed a commercially available flavonoid-rich hydroethanolic extract (Citrolive™; 1000 mg/day for 90 d) from bitter orange and olive leaf (*Olea europaea* L.), and they showed a significant reduction in ox-LDL-c and LDL-oxidase/LDL-c ratio and increased serum paraoxonase activity (PON1; athero-protective by preventing LDL oxidation) as compared to controls [124]. In another eight-week study of 96 healthy human subjects (51 intervention and 45 placeboes), supplementation of Citrolive™ (1000 mg/day for 8 weeks) improved endothelial function, as measured by flow-mediated vasodilation (FMD), reduced blood pressure and lipid metabolism-related parameters (TC, LDL-c, LDL-oxidase, oxidized/reduced glutathione (GSSH/GHS) ratio, protein carbonyl, and IL-6), and improved antioxidant and inflammatory status [125].

In a randomized, parallel, double-blind, placebo-controlled trial of 153 participants (53 women and 106 men; age 18 to 65) with pre- or stage-1 hypertensive conditions, supplementation of 500 mL/day of orange juice (containing 345 mg hesperidin) or hesperidin-enriched orange juice (containing 600 mg of hesperidin) for 12 weeks reduced systolic BP (SBP; −6.35 and −7.36 mmHg) and pulse (PP) pressure. Interestingly, the SBP and PP decreased dose-dependently relative to the hesperidin intake.

Visvanathan and Williamson [126] reviewed acute (13 studies) as well as chromic (22 studies) human intervention studies of the effect of citrus fruits and juice intake on the risk of developing type 2 diabetes and concluded that the direct acute effect of citrus polyphenols on the postprandial glycemic response (a risk factor for type 2 diabetes) is subtle. However, citrus juice intake for longer periods (e.g., 500 mL/day for 12 weeks) showed improved fasting glucose, fasting insulin (9–32%), and insulin resistance.

The lower solubility hampers the bioavailability and microbial metabolism of flavonoids, thus probably yielding high inter-individual variability, resulting in inconsistent health benefits [127]. In a randomized crossover human pharmacokinetic study, 16 healthy subjects (eight men and eight women) were administered a single dose of 3.1 g lemon extract containing 260 mg eriocitrin (main flavanone of lemon) or 1.95 g orange extract containing 260 mg hesperidin (main flavanone of orange) and showed higher bioavailability of eriocitrin, compared to hesperidin, probably due to the higher solubility of eriocitrin [127]. Thus, the authors suggested that consumption of eriocitrin-rich lemon extract could provide better health benefits.

The emerging evidence has suggested that the bioactive compounds present in orange fruits are associated with the metabolism of the gut microbiota [128]. Brasili et al. [128] revealed that daily consumption of juice of *cv*. Cara Cara and *cv*. Bahia oranges, differing in vitamin C, flavanone, and carbohydrate content, affects the fecal microbiota and metabolome differently. Intake of Cara Cara orange juice increased the Mogibacteriaceae and Tissierellaceae families (Firmicutes), while the Odoribacteraceae family and the Odoribacter genus (Bacteroidetes) decreased. In contrast, in the Bahia group, the Enterococcaceae and Veillonellaceae families increased while the Mogibacteriaceae and Ruminococcaceae families and the *Faecalibacterium prausnitzii* decreased. The abundance of Mogibacteriaceae was found in healthy subjects.

The PGs, thromboxanes (TXs), leukotrienes (LT), and isoprostane (IsoPs) metabolites derived from arachidonic acid are generally considered proinflammatory mediators, and the hallmark of increased secretion of these metabolites in the urine indicates enhanced inflammation in the body [129]. In a randomized, double-blind, placebo-controlled, and crossover study on 16 elite triathletes (6 women and 10 men), consumption of 200 mL of polyphenol-rich *Aronia*-*citrus* juice (95% citrus juice + 5% *Aronia melanocarpa* juice) for 45 d led to reduced excretion of 2,3-dinor-11β-PGF_2α_ and 11-dehydro-thromboxin B2 (11-dh-TXB2), although the levels of other metabolites related to vascular homeostasis and smooth muscle function, such as PGE2, 15-keto-15-F2t-IsoP, 20-OH-PGE2, leukotrienes E4 (LTE4), and 15-epi-15-E2t-IsoP, were increased after juice supplementation compared to the placebo, suggesting a positive effect on the cardiovascular system [129].

## 6. Toxicity and Safety Profile of Citrus Fruit Bioactive Compounds

Bioactive compounds derived from citrus fruits have shown a good safety profile in animal toxicological evaluation. In a 90 d sub-chronic and acute oral toxicity study on Sprague Dawley rats, hesperidin isolated from the dehydrated peel of *C. sinensis* showed a low observed adverse effect level (LOAEL) at 1 g/kg, and a median lethal dose (LD_50_) of 4.83 g/kg [130]. These observations suggest a good safety profile in the animals, as this concentration is much lower than the flavonoids administered in animal models (10–200 mg/kg) to obtain the health benefits (Table 4). Moreover, other citrus flavonoids, including nobiletin, tangeretin, and naringin, have shown a good safety profile [131,132].

The limonene, and other terpene-rich citrus flavor ingredients, such as oil, essential oil, whole fruit extract, and peel extract, are generally recognized as safe (GRAS) [133]. R-(+)-limonene has shown no observed adverse effect level (NOAEL) in rodents ranging from <75 to 500 mg/kg, and LD_50_ values range from 4.40 to 6.60 g/kg [134].

## 7. Conclusions

Citrus fruits are a rich and exceptional source of bioactive flavonoids, especially polymethoxylated flavones (PMFs), including nobiletin, tangeritin, and 5-demethyl nobiletin. Moreover, citrus fruits are a dense source of bioactive xanthophylls (e.g., violaxanthin esters), provitamin A carotenoids (e.g., β-cryptoxanthin), and apocarotenoids (e.g., β-citraurin). These bioactive compounds reduce the inflammatory mediators and reactive oxygen species (ROS) in the body, thus minimizing the risk of metabolic syndrome, including neurodegenerative diseases, cardiovascular disease (CVD), diabetes, and cancer. In addition, citrus essential oil, rich in terpenes (e.g., D-limonene) and limonoids (limonin), is an economically important product due to its flavoring, antimicrobial, antioxidant, and other health-beneficial properties.

Significant advancements have been made to study the composition, content, and health-promoting activities of citrus fruit bioactives. However, in future investigations, the following fields should be addressed to overcome the bottlenecks: (1) screening of traditional and new cultivars with modern analytical techniques to identify the genetic variation in content and composition; these data can help to select bioactive-rich cultivars for food formulations; moreover, precise identification of the bioactive-rich growth stage of citrus fruits suitable for consumption is necessary; (2) the elucidation of cellular and molecular mechanisms of functioning of citrus bioactives in the body; (3) more human interventional studies are required to demonstrate the health benefits of citrus bioactives; (4) the synergetic effects in the bioavailability and bioactivity among different citrus bioactive need more exploration; in addition, synergetic effects between citrus bioactives and clinically used drugs should be explored; (5) citrus fruit wastes can potentially serve as a low-cost and eco-friendly source of bioactives; however, further research is needed in the context of the efficient extraction and utilization of bioactives from citrus fruit waste.

## Figures and Tables

**Table 1:** The list of citrus fruit species widely investigated for their composition of bioactive compounds and their health benefits.

Botanical Name	Common Name
*C. aurantifolia* (Christm.) Swingle or *C. × lumia* Risso. & Poit.	Lime, key lime, lumy, ancient Mediterranean citrus
*C. × aurantium* L.	Bitter/sour orange
*C. × clementina*	Clementine
*C. × deliciosa* Tanore	Montenegrin mandarin
*C. japonica* Thunb.	Kumquat
*C. junos* Siebold ex Tanaka	Junos, yuzu
*C. × latifolia* (Yu.Tanaka) Tanaka	Persian lime
*C. limon* (L.) Osbeck)	Lemon
*C. × limonia* Osbeck	Rangpur lime
*C. maxima* (J. Burman) Merr. or *C. grandis* (L.) Osbeck	Pomelo, pummelo
*C. medica* L.	Citron, finger citron
*C. poonensis* Hort. ex Tanaka	Ponkan
*C. × paradisi* Macfad. or *C. × paradise* Macfad	Grapefruit, pink/white grapefruit
*C. reticulata* × *C. paradisi*	Tangelo
*C. reticulata* Blanco	Mandarin, tangerine, Phlegraean mandarin, ougan
*C. reticulata* × *C. sinensis*	Tangor
*C. × sinensis* (L.) Osbeck	Orange, Valencia orange, blood orange, sweet orange
*C. unshiu* Marc.	Satsuma mandarin, Mandarin orange

**Table 2:** List of recently published outstanding reviews on composition and health benefits of citrus bioactive compounds.

Compounds	Review Highlights	Reference
Essential oil	Extraction, purification, detection methods, composition, and applications of citrus essential oil	[10]
	Composition of volatile compounds from peel, leaves, and flowers of different citrus species	[11]
Flavanones (hesperidin and naringin)	The intestinal fate, bioavailability, intestinal metabolism, and interaction with the gut microbiota	[12]
Flavones	Sources, antioxidant, anti-inflammatory, antimicrobial, anticancer properties	[13]
Flavonoids	Chemistry, biosynthesis, composition, extraction techniques, health benefits, and industrial applications	[14]
	Composition, antioxidant evaluation, and regulation of Nrf2-Keap1 pathway by citrus flavonoids	[15]
	Role of citrus flavonoids in brain health: evidence from preclinical and human studies	[16]
	Biosynthesis, location, and distribution of flavonoids in citrus plants, factors affecting biosynthesis, and health-promoting properties	[17]
	In vitro, in vivo, and human studies of citrus flavonoids in minimizing the incidence of inflammatory bowel disease	[18]
	Antidiabetic potential of 19 citrus flavonoids, including diosmin, hesperidin, hesperetin, naringin, naringenin, nobiletin, neohesperidin, quercetin, rutin, and tangeretin	[19]
	Therapeutic potential in diabetes and diabetic cardiomyopathy, endothelial dysfunction, atherosclerosis, and platelet function	[20]
	Chemistry, metabolism, bioavailability, biotransformation and delivery systems, and health benefits	[6]
Hesperidin and vitamin C	Antiviral properties against acute respiratory syndrome coronavirus 2 (SARS-CoV-2)	[21]
Naringenin	Antidiabetic properties; in vitro, in vivo, and human studies	[22]
	Combating oxidative stress disorders: cardiovascular disease, diabetes mellitus, neurodegenerative disease, pulmonary disease, cancer, and nephropathy	[23]
Nobiletin	Beneficial effects against Alzheimer’s disease (AD) and Parkinson’s disease (PD)	[24]
Nobiletin, 5-demethylnobiletin, and derivatives	Beneficial effects against colon cancer, pharmacokinetics, and bioavailability	[25]
Nutrients and bioactive	Description of the genus *Citrus*, the composition of nutrients and bioactive components, and biological activities of lemon extract and essential oil	[26]
	Nutrients (proteins, lipids, vitamins, minerals, fiber) and bioactive (flavonoids, essential oil, limonoids, carotenoids, synephrine) content, their structural characteristics, and health benefits	[5]
Polymethoxyflavones (PMF)	Biological properties against metabolic disorder, atherosclerosis, inflammation, neuroinflammation, cancer, and oxidation	[27]

## Data Availability

Data are contained within the article.

## References

[1] Lapuente M., Estruch R., Shahbaz M., Casas R. (2019). Relation of Fruits and Vegetables with Major Cardiometabolic Risk Factors, Markers of Oxidation, and Inflammation. Nutrients.

[2] Medina-Remón A., Kirwan R., Lamuela-Raventós R.M., Estruch R. (2018). Dietary patterns and the risk of obesity, type 2 diabetes mellitus, cardiovascular diseases, asthma, and neurodegenerative diseases. Crit. Rev. Food Sci. Nutr..

[3] Zou Z., Xi W., Hu Y., Nie C., Zhou Z. (2016). Antioxidant activity of Citrus fruits. Food Chem..

[4] Wu G.A., Terol J., Ibanez V., Lopez-Garcia A., Perez-Roman E., Borreda C., Domingo C., Tadeo F.R., Carbonell-Caballero J., Alonso R. (2018). Genomics of the origin and evolution of Citrus. Nature.

[5] Lu X., Zhao C., Shi H., Liao Y., Xu F., Du H., Xiao H., Zheng J. (2021). Nutrients and bioactives in citrus fruits: Different citrus varieties, fruit parts, and growth stages. Crit. Rev. Food Sci. Nutr..

[6] Zhang M., Zhu S.Y., Yang W.J., Huang Q.R., Ho C.T. (2021). The biological fate and bioefficacy of citrus flavonoids: Bioavailability, biotransformation, and delivery systems. Food Funct..

[7] United States Department of Agriculture Foreign Agricultural Service (2021). Citrus: World Markets and Trade. https://www.fas.usda.gov/data/citrus-world-markets-and-trade.

[8] Cebadera-Miranda L., Dominguez L., Dias M.I., Barros L., Ferreira I., Igual M., Martinez-Navarrete N., Fernandez-Ruiz V., Morales P., Camara M. (2019). Sanguinello and Tarocco (*Citrus sinensis* L. Osbeck): Bioactive compounds and colour appearance of blood oranges. Food Chem..

[9] Lado J., Gambetta G., Zacarias L. (2018). Key determinants of citrus fruit quality: Metabolites and main changes during maturation. Sci. Hortic..

[10] Bora H., Kamle M., Mahato D.K., Tiwari P., Kumar P. (2020). Citrus Essential Oils (CEOs) and Their Applications in Food: An Overview. Plants.

[11] Gonzalez-Mas M.C., Rambla J.L., Lopez-Gresa M.P., Blazquez M.A., Granell A. (2019). Volatile Compounds in Citrus Essential Oils: A Comprehensive Review. Front. Plant Sci..

[12] Stevens Y., Van Rymenant E., Grootaert C., Van Camp J., Possemiers S., Masclee A., Jonkers D. (2019). The Intestinal Fate of Citrus Flavanones and Their Effects on Gastrointestinal Health. Nutrients.

[13] Barreca D., Mandalari G., Calderaro A., Smeriglio A., Trombetta D., Felice M.R., Gattuso G. (2020). Citrus Flavones: An Update on Sources, Biological Functions, and Health Promoting Properties. Plants.

[14] Addi M., Elbouzidi A., Abid M., Tungmunnithum D., Elamrani A., Hano C. (2022). An Overview of Bioactive Flavonoids from Citrus Fruits. Appl. Sci..

[15] Wang Y., Liu X.J., Chen J.B., Cao J.P., Li X., Sun C.D. (2021). Citrus flavonoids and their antioxidant evaluation. Crit. Rev. Food Sci. Nutr..

[16] Pontifex M.G., Malik M., Connell E., Muller M., Vauzour D. (2021). Citrus Polyphenols in Brain Health and Disease: Current Perspectives. Front. Neurosci..

[17] Zhao C.Y., Wang F., Lian Y.H., Xiao H., Zheng J.K. (2020). Biosynthesis of citrus flavonoids and their health effects. Crit. Rev. Food Sci. Nutr..

[18] Musumeci L., Maugeri A., Cirmi S., Lombardo G.E., Russo C., Gangemi S., Calapai G., Navarra M. (2020). Citrus fruits and their flavonoids in inflammatory bowel disease: An overview. Nat. Prod. Res..

[19] Gandhi G.R., Vasconcelos A.B.S., Wu D.T., Li H.B., Antony P.J., Li H., Geng F., Gurgel R.Q., Narain N., Gan R.Y. (2020). Citrus Flavonoids as Promising Phytochemicals Targeting Diabetes and Related Complications: A Systematic Review of In Vitro and In Vivo Studies. Nutrients.

[20] Mahmoud A.M., Bautista R.J.H., Sandhu M.A., Hussein O.E. (2019). Beneficial Effects of Citrus Flavonoids on Cardiovascular and Metabolic Health. Oxid. Med. Cell. Longev..

[21] Bellavite P., Donzelli A. (2020). Hesperidin and SARS-CoV-2: New Light on the Healthy Function of Citrus Fruits. Antioxidants.

[22] Den Hartogh D.J., Tsiani E. (2019). Antidiabetic Properties of Naringenin: A Citrus Fruit Polyphenol. Biomolecules.

[23] Zaidun N.H., Thent Z.C., Abd Latiff A. (2018). Combating oxidative stress disorders with citrus flavonoid: Naringenin. Life Sci..

[24] Nakajima A., Ohizumi Y. (2019). Potential Benefits of Nobiletin, A Citrus Flavonoid, against Alzheimer’s Disease and Parkinson’s Disease. Int. J. Mol. Sci..

[25] Goh J.X.H., Tan L.T.H., Goh J.K., Chan K.G., Pusparajah P., Lee L.H., Goh B.H. (2019). Nobiletin and Derivatives: Functional Compounds from Citrus Fruit Peel for Colon Cancer Chemoprevention. Cancers.

[26] Klimek-Szczykutowicz M., Szopa A., Ekiert H. (2020). *Citrus limon* (Lemon) Phenomenon—A Review of the Chemistry, Pharmacological Properties, Applications in the Modern Pharmaceutical, Food, and Cosmetics Industries, and Biotechnological Studies. Plants.

[27] Gao Z., Gao W., Zeng S.L., Li P., Liu E.H. (2018). Chemical structures, bioactivities and molecular mechanisms of citrus polymethoxyflavones. J. Funct. Foods.

[28] Peng Z.X., Zhang H.P., Li W.Y., Yuan Z.Y., Xie Z.Z., Zhang H.Y., Cheng Y.J., Chen J.J., Xu J. (2021). Comparative profiling and natural variation of polymethoxylated flavones in various citrus germplasms. Food Chem..

[29] Zhang H.J., Tian G.F., Zhao C.Y., Han Y.H., DiMarco-Crook C., Lu C., Bao Y.M., Li C.X., Xiao H., Zheng J.K. (2019). Characterization of polymethoxyflavone demethylation during drying processes of citrus peels. Food Funct..

[30] Smeriglio A., Cornara L., Denaro M., Barreca D., Burlando B., Xiao J.B., Trombetta D. (2019). Antioxidant and cytoprotective activities of an ancient Mediterranean citrus (*Citrus lumia* Risso) albedo extract: Microscopic observations and polyphenol characterization. Food Chem..

[31] Mazzotti F., Bartella L., Talarico I.R., Napoli A., Di Donna L. (2021). High-throughput determination of flavanone-O-glycosides in citrus beverages by paper spray tandem mass spectrometry. Food Chem..

[32] Rong X., Xu J., Jiang Y., Li F., Chen Y.L., Dou Q.P., Li D.P. (2021). Citrus peel flavonoid nobiletin alleviates lipopolysaccharide-induced inflammation by activating IL-6/STAT3/FOXO3a-mediated autophagy. Food Funct..

[33] Wang F., Zhao C.Y., Yang M.K., Zhang L., Wei R.J., Meng K., Bao Y.M., Zhang L.N., Zheng J.K. (2021). Four Citrus Flavanones Exert Atherosclerosis Alleviation Effects in ApoE(−/−) Mice via Different Metabolic and Signaling Pathways. J. Agric. Food Chem..

[34] Zhang M., Zhu J.Y., Zhang X., Zhao D.G., Ma Y.Y., Li D.L., Ho C.T., Huang Q.R. (2020). Aged citrus peel (chenpi) extract causes dynamic alteration of colonic microbiota in high-fat diet induced obese mice. Food Funct..

[35] Zeng S.L., Li S.Z., Xiao P.T., Cai Y.Y., Chu C., Chen B.Z., Li P., Li J., Liu E.H. (2020). Citrus polymethoxyflavones attenuate metabolic syndrome by regulating gut microbiome and amino acid metabolism. Sci. Adv..

[36] Song M.Y., Lan Y.Q., Wu X., Han Y.H., Wang M.Q., Zheng J.K., Li Z.Z., Li F., Zhou J.Z., Xiao J. (2020). The chemopreventive effect of 5-demethylnobiletin, a unique citrus flavonoid, on colitis-driven colorectal carcinogenesis in mice is associated with its colonic metabolites. Food Funct..

[37] Li S., Pan M.-H., Lo C.-Y., Tan D., Wang Y., Shahidi F., Ho C.-T. (2009). Chemistry and health effects of polymethoxyflavones and hydroxylated polymethoxyflavones. J. Funct. Foods.

[38] Deng M., Jia X.C., Dong L.H., Liu L., Huang F., Chi J.W., Ma Q., Zhao D., Zhang M.W., Zhang R.F. (2022). Structural elucidation of flavonoids from Shatianyu (*Citrus grandis* L. Osbeck) pulp and screening of key antioxidant components. Food Chem..

[39] Chen J., Yuan Z., Zhang H., Li W., Shi M., Peng Z., Li M., Tian J., Deng X., Cheng Y. (2019). Cit1,2RhaT and two novel CitdGlcTs participate in flavor-related flavonoid metabolism during citrus fruit development. J. Exp. Bot..

[40] Ledesma-Escobar C.A., Priego-Capote F., Olvera V.J.R., de Castro M.D.L. (2018). Targeted Analysis of the Concentration Changes of Phenolic Compounds in Persian Lime (*Citrus latifolia*) during Fruit Growth. J. Agric. Food Chem..

[41] Multari S., Licciardello C., Caruso M., Martens S. (2020). Monitoring the changes in phenolic compounds and carotenoids occurring during fruit development in the tissues of four citrus fruits. Food Res. Int..

[42] Saini R.K., Nile S.H., Park S.W. (2015). Carotenoids from fruits and vegetables: Chemistry, analysis, occurrence, bioavailability and biological activities. Food Res. Int..

[43] Saini R.K., Keum Y.S. (2018). Carotenoid extraction methods: A review of recent developments. Food Chem..

[44] Etzbach L., Stolle R., Anheuser K., Herdegen V., Schieber A., Weber F. (2020). Impact of Different Pasteurization Techniques and Subsequent Ultrasonication on the In Vitro Bioaccessibility of Carotenoids in Valencia Orange (*Citrus sinensis* (L.) Osbeck) Juice. Antioxidants.

[45] Lux P.E., Carle R., Zacarias L., Rodrigo M.J., Schweiggert R.M., Steingass C.B. (2019). Genuine Carotenoid Profiles in Sweet Orange *Citrus sinensis* (L.) Osbeck cv. Navel Peel and Pulp at Different Maturity Stages. J. Agric. Food Chem..

[46] Zheng X.J., Mi J.N., Deng X.X., Al-Babili S. (2021). LC-MS-Based Profiling Provides New Insights into Apocarotenoid Biosynthesis and Modifications in Citrus Fruits. J. Agric. Food Chem..

[47] Luan Y.T., Wang S.S., Wang R.Q., Xu C.J. (2020). Accumulation of red apocarotenoid beta-citraurin in peel of a spontaneous mutant of huyou (*Citrus changshanensis*) and the effects of storage temperature and ethylene application. Food Chem..

[48] Dhakane-Lad J., Kar A. (2021). Supercritical CO2 extraction of lycopene from pink grapefruit (*Citrus paradise* Macfad) and its degradation studies during storage. Food Chem..

[49] Wang F., Lin J.R., Xu L.L., Peng Q.Q., Huang H.Y., Tong L.J., Lu Q., Wang C.C., Yang L. (2019). On higher nutritional and medical properties of a carotenoid-rich mutant pomelo (*Citrus maxima* (L.) Osbeck). Ind. Crop. Prod..

[50] Petry F.C., de Nadai F.B., Cristofani-Yaly M., Latado R.R., Mercadante A.Z. (2019). Carotenoid biosynthesis and quality characteristics of new hybrids between tangor (*Citrus reticulata* × *C. sinensis*) cv. ‘Murcott’ and sweet orange (*C. sinensis*) cv. ‘Pera’. Food Res. Int..

[51] Raspo M.A., Vignola M.B., Andreatta A.E., Juliani H.R. (2020). Antioxidant and antimicrobial activities of citrus essential oils from Argentina and the United States. Food Biosci..

[52] Gao Z.P., Zhong W.M., Chen K.Y., Tang P.Y., Guo J.J. (2020). Chemical composition and anti-biofilm activity of essential oil from *Citrus medica* L. var. sarcodactylis Swingle against Listeria monocytogenes. Ind Crop. Prod..

[53] Mahato N., Sharma K., Koteswararao R., Sinha M., Baral E., Cho M.H. (2019). Citrus essential oils: Extraction, authentication and application in food preservation. Crit. Rev. Food Sci. Nutr..

[54] Li Z.H., Cai M., Liu Y.S., Sun P.L., Luo S.L. (2019). Antibacterial Activity and Mechanisms of Essential Oil from *Citrus medica* L. var. sarcodactylis. Molecules.

[55] Ambrosio C.M.S., Ikeda N.Y., Miano A.C., Saldana E., Moreno A.M., Stashenko E., Contreras-Castillo C.J., Da Gloria E.M. (2019). Unraveling the selective antibacterial activity and chemical composition of citrus essential oils. Sci. Rep..

[56] Denkova-Kostova R., Teneva D., Tomova T., Goranov B., Denkova Z., Shopska V., Slavchev A., Hristova-Ivanova Y. (2021). Chemical composition, antioxidant and antimicrobial activity of essential oils from tangerine (*Citrus reticulata* L.), grapefruit (*Citrus paradisi* L.), lemon (*Citrus lemon* L.) and cinnamon (*Cinnamomum zeylanicum* Blume). Z. Nat. Sect. C J. Biosci..

[57] Paw M., Begum T., Gogoi R., Pandey S.K., Lal M. (2020). Chemical Composition of *Citrus limon* L. Burmf Peel Essential Oil from North East India. J. Essent. Oil Bear. Plants.

[58] Zoccali M., Arigo A., Russo M., Salafia F., Dugo P., Mondello L. (2018). Characterization of Limonoids in Citrus Essential Oils by Means of Supercritical Fluid Chromatography Tandem Mass Spectrometry. Food Anal. Methods.

[59] Rossi R.C., da Rosa S.R., Weimer P., Moura J.G.L., de Oliveira V.R., de Castilhos J. (2020). Assessment of compounds and cytotoxicity of *Citrus deliciosa* Tenore essential oils: From an underexploited by-product to a rich source of high-value bioactive compounds. Food Biosci..

[60] Yu M., Xia Y., Xie W., Li Y., Yu X., Zheng J., Zhang Y. (2021). Enzymatic extraction of pectic oligosaccharides from finger citron (*Citrus medica* L. var. *sarcodactylis* Swingle) pomace with antioxidant potential. Food Funct..

[61] Zema D.A., Calabro P.S., Folino A., Tamburino V., Zappia G., Zimbone S.M. (2018). Valorisation of citrus processing waste: A review. Waste Manag..

[62] Mahato N., Sharma K., Sinha M., Cho M.H. (2018). Citrus waste derived nutra-/pharmaceuticals for health benefits: Current trends and future perspectives. J. Funct. Foods.

[63] Singh B., Singh J.P., Kaur A., Yadav M.P. (2021). Insights into the chemical composition and bioactivities of citrus peel essential oils. Food Res. Int..

[64] Saini A., Panesar P.S., Bera M.B. (2021). Valuation of *Citrus reticulata* (kinnow) peel for the extraction of lutein using ultrasonication technique. Biomass Convers. Biorefinery.

[65] Costanzo G., Iesce M.R., Naviglio D., Ciaravolo M., Vitale E., Arena C. (2020). Comparative Studies on Different Citrus Cultivars: A Revaluation of Waste Mandarin Components. Antioxidants.

[66] Murador D.C., Salafia F., Zoccali M., Martins P.L.G., Ferreira A.G., Dugo P., Mondello L., De Rosso V.V., Giuffrida D. (2019). Green Extraction Approaches for Carotenoids and Esters: Characterization of Native Composition from Orange Peel. Antioxidants.

[67] Nuzzo D., Picone P., Giardina C., Scordino M., Mudò G., Pagliaro M., Scurria A., Meneguzzo F., Ilharco L.M., Fidalgo A. (2021). New Neuroprotective Effect of Lemon IntegroPectin on Neuronal Cellular Model. Antioxidants.

[68] Colodel C., Vriesmann L.C., Teofilo R.F., Petkowicz C.L.D. (2018). Extraction of pectin from ponkan (*Citrus reticulata* Blanco cv. Ponkan) peel: Optimization and structural characterization. Int. J. Biol. Macromol..

[69] Long X.Y., Zeng X.G., Yan H.T., Xu M.J., Zeng Q.T., Xu C., Xu Q.M., Liang Y., Zhang J. (2021). Flavonoids composition and antioxidant potential assessment of extracts from Gannanzao Navel Orange (*Citrus sinensis* Osbeck Cv. Gannanzao) peel. Nat. Prod. Res..

[70] El-Kersh D.M., Ezzat S.M., Salama M.M., Mahrous E.A., Attia Y.M., Ahmed M.S., Elmazar M.M. (2021). Anti-estrogenic and anti-aromatase activities of citrus peels major compounds in breast cancer. Sci. Rep..

[71] Chen Y., Pan H.L., Hao S.X., Pan D.M., Wang G.J., Yu W.Q. (2021). Evaluation of phenolic composition and antioxidant properties of different varieties of Chinese citrus. Food Chem..

[72] Liu N., Li X., Zhao P., Zhang X.Q., Qiao O., Huang L.Q., Guo L.P., Gao W.Y. (2021). A review of chemical constituents and health-promoting effects of citrus peels. Food Chem..

[73] Gogoi M., Boruah J.L.H., Bora P.K., Das D.J., Famhawite V., Biswas A., Puro N., Kalita J., Haldar S., Baishya R. (2021). Citrus macroptera induces apoptosis via death receptor and mitochondrial mediated pathway as prooxidant in human non-small cell lung cancer cells. Food Biosci..

[74] Abdelghffar E.A., El-Nashar H.A.S., Al-Mohammadi A.G.A., Eldahshan O.A. (2021). Orange fruit (*Citrus sinensis*) peel extract attenuates chemotherapy-induced toxicity in male rats. Food Funct..

[75] Singh B., Singh J.P., Kaur A., Singh N. (2020). Phenolic composition, antioxidant potential and health benefits of citrus peel. Food Res. Int..

[76] Jeong D., Park H., Jang B.K., Ju Y.B., Shin M.H., Oh E.J., Lee E.J., Kim S.R. (2021). Recent advances in the biological valorization of citrus peel waste into fuels and chemicals. Bioresour. Technol..

[77] Zayed A., Badawy M.T., Farag M.A. (2021). Valorization and extraction optimization of Citrus seeds for food and functional food applications. Food Chem..

[78] Hwang H.J., Kim H.J., Ko M.J., Chung M.S. (2021). Recovery of hesperidin and narirutin from waste *Citrus unshiu* peel using subcritical water extraction aided by pulsed electric field treatment. Food Sci. Biotechnol..

[79] Kim D.-S., Lim S.-B. (2020). Semi-Continuous Subcritical Water Extraction of Flavonoids from *Citrus unshiu* Peel: Their Antioxidant and Enzyme Inhibitory Activities. Antioxidants.

[80] Kim J.W., Jo E.H., Moon J.E., Cha H., Chang M.H., Cho H.T., Lee M.K., Jung W.S., Lee J.H., Heo W. (2020). In Vitro and In Vivo Inhibitory Effect of Citrus Junos Tanaka Peel Extract against Oxidative Stress-Induced Apoptotic Death of Lung Cells. Antioxidants.

[81] Anacleto S.L., Milenkovic D., Kroon P.A., Needs P.W., Lajolo F.M., Hassimotto N.M.A. (2020). Citrus flavanone metabolites protect pancreatic-beta cells under oxidative stress induced by cholesterol. Food Funct..

[82] Huang R., Zhang Y., Shen S.Y., Zhi Z.J., Cheng H., Chen S.G., Ye X.Q. (2020). Antioxidant and pancreatic lipase inhibitory effects of flavonoids from different citrus peel extracts: An in vitro study. Food Chem..

[83] Denaro M., Smeriglio A., Trombetta D. (2021). Antioxidant and Anti-Inflammatory Activity of Citrus Flavanones Mix and Its Stability after In Vitro Simulated Digestion. Antioxidants.

[84] Yang W.L., Chen S.Y., Ho C.Y., Yen G.C. (2020). Citrus flavonoids suppress IL-5 and ROS through distinct pathways in PMA/ionomycin-induced EL-4 cells. Food Funct..

[85] Hu H.J., Zhang S.S., Pan S.Y. (2021). Characterization of Citrus Pectin Oligosaccharides and Their Microbial Metabolites as Modulators of Immunometabolism on Macrophages. J. Agric. Food Chem..

[86] Smeriglio A., Alloisio S., Raimondo F.M., Denaro M., Xiao J.B., Cornara L., Trombetta D. (2018). Essential oil of *Citrus lumia* Risso: Phytochemical profile, antioxidant properties and activity on the central nervous system. Food Chem. Toxicol..

[87] Piccialli I., Tedeschi V., Caputo L., Amato G., De Martino L., De Feo V., Secondo A., Pannaccione A. (2021). The Antioxidant Activity of Limonene Counteracts Neurotoxicity Triggered byAβ1-42 Oligomers in Primary Cortical Neurons. Antioxidants.

[88] Cirmi S., Maugeri A., Lombardo G.E., Russo C., Musumeci L., Gangemi S., Calapai G., Barreca D., Navarra M. (2021). A Flavonoid-Rich Extract of Mandarin Juice Counteracts 6-OHDA-Induced Oxidative Stress in SH-SY5Y Cells and Modulates Parkinson-Related Genes. Antioxidants.

[89] Kitagawa T., Matsumoto T., Imahori D., Kobayashi M., Okayama M., Ohta T., Yoshida T., Watanabe T. (2021). Limonoids isolated from the Fortunella crassifolia and the *Citrus junos* with their cell death-inducing activity on Adriamycin-treated cancer cell. J. Nat. Med..

[90] Murthy K.N.C., Jayaprakasha G.K., Safe S., Patil B.S. (2021). Citrus limonoids induce apoptosis and inhibit the proliferation of pancreatic cancer cells. Food Funct..

[91] Dong W., Wei X., Zhang F., Hao J., Huang F., Zhang C., Liang W. (2014). A dual character of flavonoids in influenza A virus replication and spread through modulating cell-autonomous immunity by MAPK signaling pathways. Sci. Rep..

[92] Lin C.W., Tsai F.J., Tsai C.H., Lai C.C., Wan L., Ho T.Y., Hsieh C.C., Chao P.D. (2005). Anti-SARS coronavirus 3C-like protease effects of Isatis indigotica root and plant-derived phenolic compounds. Antivir. Res..

[93] De Clercq E. (2006). Potential antivirals and antiviral strategies against SARS coronavirus infections. Expert Rev. Anti-Infect. Ther..

[94] Tutunchi H., Naeini F., Ostadrahimi A., Hosseinzadeh-Attar M.J. (2020). Naringenin, a flavanone with antiviral and anti-inflammatory effects: A promising treatment strategy against COVID-19. Phytother. Res..

[95] Ferreira S.S., Silva A.M., Nunes F.M. (2018). *Citrus reticulata* Blanco peels as a source of antioxidant and anti-proliferative phenolic compounds. Ind. Crop. Prod..

[96] Saini R.K., Rengasamy K.R.R., Mahomoodally F.M., Keum Y.S. (2020). Protective effects of lycopene in cancer, cardiovascular, and neurodegenerative diseases: An update on epidemiological and mechanistic perspectives. Pharm. Res..

[97] Saini R.K., Keum Y.S., Daglia M., Rengasamy K.R. (2020). Dietary carotenoids in cancer chemoprevention and chemotherapy: A review of emerging evidence. Pharm. Res..

[98] Shin J., Song M.H., Oh J.W., Keum Y.S., Saini R.K. (2020). Pro-Oxidant Actions of Carotenoids in Triggering Apoptosis of Cancer Cells: A Review of Emerging Evidence. Antioxidants.

[99] Tian D.M., Wang F.F., Duan M.L., Cao L.Y., Zhang Y.W., Yao X.S., Tang J.S. (2019). Coumarin Analogues from the *Citrus grandis* (L.) Osbeck and Their Hepatoprotective Activity. J. Agric. Food Chem..

[100] Cataneo A.H.D., Kuczera D., Koishi A.C., Zanluca C., Silveira G.F., de Arruda T.B., Suzukawa A.A., Bortot L.O., Dias-Baruffi M., Verri W.A. (2019). The citrus flavonoid naringenin impairs the in vitro infection of human cells by Zika virus. Sci. Rep..

[101] Gansukh E., Nile A., Sivanesan I., Rengasamy K.R.R., Kim D.H., Keum Y.S., Saini R.K. (2019). Chemopreventive Effect of beta-Cryptoxanthin on Human Cervical Carcinoma (HeLa) Cells Is Modulated through Oxidative Stress-Induced Apoptosis. Antioxidants.

[102] Feng K.L., Zhu X.A., Liu G., Kan Q.X., Chen T., Chen Y.J., Cao Y. (2020). Dietary citrus peel essential oil ameliorates hypercholesterolemia and hepatic steatosis by modulating lipid and cholesterol homeostasis. Food Funct..

[103] Dhuique-Mayer C., Gence L., Portet K., Tousch D., Poucheret P. (2020). Preventive action of retinoids in metabolic syndrome/type 2 diabetic rats fed with citrus functional food enriched in beta-cryptoxanthin. Food Funct..

[104] Revathy J., Srinivasan S., Abdullah S.H.S., Muruganathan U. (2018). Antihyperglycemic effect of hesperetin, a citrus flavonoid, extenuates hyperglycemia and exploring the potential role in antioxidant and antihyperlipidemic in streptozotocin-induced diabetic rats. Biomed. Pharmacother..

[105] Bi W.Y., Zhou J.X., Zhao L., Wang C.T., Wu W., Zhang L.B., Ji B.P., Zhang N.H., Zhou F. (2021). Preventive effect of different citrus essential oils on primary dysmenorrhea: In vivo and in vitro study. Food Biosci..

[106] Zhang L.L., Yang Z.Y., Fan G., Ren J.N., Yin K.J., Pan S.Y. (2019). Antidepressant-like Effect of *Citrus sinensis* (L.) Osbeck Essential Oil and Its Main Component Limonene on Mice. J. Agric. Food Chem..

[107] Muhammad T., Ikram M., Ullah R., Rehman S.U., Kim M.O. (2019). Hesperetin, a Citrus Flavonoid, Attenuates LPS-Induced Neuroinflammation, Apoptosis and Memory Impairments by Modulating TLR4/NF-kappa B Signaling. Nutrients.

[108] Okuyama S., Nakashima T., Nakamura K., Shinoka W., Kotani M., Sawamoto A., Nakajima M., Furukawa Y. (2018). Inhibitory Effects of Auraptene and Naringin on Astroglial Activation, Tau Hyperphosphorylation, and Suppression of Neurogenesis in the Hippocampus of Streptozotocin-Induced Hyperglycemic Mice. Antioxidants.

[109] Guo X., Cao X.D., Fang X.G., Guo A.L., Li E.H. (2021). Inhibitory effects of fermented Ougan (*Citrus reticulata* cv. *Suavissima*) juice on high-fat diet-induced obesity associated with white adipose tissue browning and gut microbiota modulation in mice. Food Funct..

[110] Lazar V., Ditu L.-M., Pircalabioru G.G., Picu A., Petcu L., Cucu N., Chifiriuc M.C. (2019). Gut Microbiota, Host Organism, and Diet Trialogue in Diabetes and Obesity. Front. Nutr..

[111] Tung Y.C., Chang W.T., Li S.M., Wu J.C., Badmeav V., Ho C.T., Pan M.H. (2018). Citrus peel extracts attenuated obesity and modulated gut microbiota in mice with high-fat diet-induced obesity. Food Funct..

[112] Miler M., Zivanovic J., Ajdzanovic V., Milenkovic D., Jaric I., Sosic-Jurjevic B., Milosevic V. (2020). Citrus Flavanones Upregulate Thyrotroph Sirt1 and Differently Affect Thyroid Nrf2 Expressions in Old-Aged Wistar Rats. J. Agric. Food Chem..

[113] Kawabata A., Hung T.V., Nagata Y., Fukuda N., Suzuki T. (2018). Citrus kawachiensis Peel Powder Reduces Intestinal Barrier Defects and Inflammation in Colitic Mice. J. Agric. Food Chem..

[114] Zhang Z.Y., Nie M.M., Liu C.Q., Jiang N., Liu C.J., Li D.J. (2019). Citrus Flavanones Enhance beta-Carotene Uptake in Vitro Experiment Using Caco-2 Cell: Structure-Activity Relationship and Molecular Mechanisms. J. Agric. Food Chem..

[115] Castro M.A., Rodenak-Kladniew B., Massone A., Polo M., de Bravo M.G., Crespo R. (2018). Citrus reticulata peel oil inhibits non-small cell lung cancer cell proliferation in culture and implanted in nude mice. Food Funct..

[116] Gao X.G., Wang C.Y., Ning C.Q., Liu K.X., Wang X.Y., Liu Z.H., Sun H.J., Ma X.D., Sun P.Y., Meng Q. (2018). Hepatoprotection of auraptene from peels of citrus fruits against thioacetamide-induced hepatic fibrosis in mice by activating farnesoid X receptor. Food Funct..

[117] Wang J.Q., Fu T., Dong R.C., Wang C.Y., Liu K.X., Sun H.J., Huo X.K., Ma X.D., Yang X.B., Meng Q. (2019). Hepatoprotection of auraptene from the peels of citrus fruits against 17 alpha-ethinylestradiol-induced cholestasis in mice by activating farnesoid X receptor. Food Funct..

[118] Carvalho J.D., Ramadan D., Goncalves V.D., Maquera-Huacho P.M., Assis R.P., Lima T.F.O., Brunetti I.L., Spolidorio D.M.P., Cesar T., Manthey J.A. (2021). Impact of citrus flavonoid supplementation on inflammation in lipopolysaccharide-induced periodontal disease in mice. Food Funct..

[119] He W., Li Y.M., Liu M.Y., Yu H.Y., Chen Q., Chen Y., Ruan J.Y., Ding Z.J., Zhang Y., Wang T. (2018). Citrus aurantium L. and Its Flavonoids Regulate TNBS-Induced Inflammatory Bowel Disease through Anti-Inflammation and Suppressing Isolated Jejunum Contraction. Int. J. Mol. Sci..

[120] Samie A., Sedaghat R., Baluchnejadmojarad T., Roghani M. (2018). Hesperetin, a citrus flavonoid, attenuates testicular damage in diabetic rats via inhibition of oxidative stress, inflammation, and apoptosis. Life Sci..

[121] Guirro M., Gual-Grau A., Gibert-Ramos A., Alcaide-Hidalgo J.M., Canela N., Arola L., Mayneris-Perxachs J. (2020). Metabolomics Elucidates Dose-Dependent Molecular Beneficial Effects of Hesperidin Supplementation in Rats Fed an Obesogenic Diet. Antioxidants.

[122] Hu H.J., Zhang S.S., Liu F.X., Zhang P.P., Muhammad Z., Pan S.Y. (2019). Role of the Gut Microbiota and Their Metabolites in Modulating the Cholesterol-Lowering Effects of Citrus Pectin Oligosaccharides in C57BL/6 Mice. J. Agric. Food Chem..

[123] Cirmi S., Navarra M., Woodside J.V., Cantwell M.M. (2018). Citrus fruits intake and oral cancer risk: A systematic review and meta-analysis. Pharmacol. Res..

[124] Victoria-Montesinos D., Abellán Ruiz M.S., Luque Rubia A.J., Guillén Martínez D., Pérez-Piñero S., Sánchez Macarro M., García-Muñoz A.M., Cánovas García F., Castillo Sánchez J., López-Román F.J. (2021). Effectiveness of Consumption of a Combination of Citrus Fruit Flavonoids and Olive Leaf Polyphenols to Reduce Oxidation of Low-Density Lipoprotein in Treatment-Naïve Cardiovascular Risk Subjects: A Randomized Double-Blind Controlled Study. Antioxidants.

[125] Sánchez Macarro M., Martínez Rodríguez J.P., Bernal Morell E., Pérez-Piñero S., Victoria-Montesinos D., García-Muñoz A.M., Cánovas García F., Castillo Sánchez J., López-Román F.J. (2020). Effect of a Combination of Citrus Flavones and Flavanones and Olive Polyphenols for the Reduction of Cardiovascular Disease Risk: An Exploratory Randomized, Double-Blind, Placebo-Controlled Study in Healthy Subjects. Nutrients.

[126] Visvanathan R., Williamson G. (2021). Effect of citrus fruit and juice consumption on risk of developing type 2 diabetes: Evidence on polyphenols from epidemiological and intervention studies. Trends Food Sci. Technol..

[127] Ávila-Gálvez M.Á., Giménez-Bastida J.A., González-Sarrías A., Espín J.C. (2021). New Insights into the Metabolism of the Flavanones Eriocitrin and Hesperidin: A Comparative Human Pharmacokinetic Study. Antioxidants.

[128] Brasili E., Hassimotto N.M.A., Del Chierico F., Marini F., Quagliariello A., Sciubba F., Miccheli A., Putignani L., Lajolo F. (2019). Daily Consumption of Orange Juice from *Citrus sinensis* L. Osbeck cv. Cara Cara and cv. Bahia Differently Affects Gut Microbiota Profiling as Unveiled by an Integrated Meta-Omits Approach. J. Agric. Food Chem..

[129] Garcia-Flores L.A., Medina S., Gomez C., Wheelock C.E., Cejuela R., Martinez-Sanz J.M., Oger C., Galano J.M., Durand T., Hernandez-Saez A. (2018). Aronia-citrus juice (polyphenol-rich juice) intake and elite triathlon training: A lipidomic approach using representative oxylipins in urine. Food Funct..

[130] Li Y., Kandhare A.D., Mukherjee A.A., Bodhankar S.L. (2019). Acute and sub-chronic oral toxicity studies of hesperidin isolated from orange peel extract in Sprague Dawley rats. Regul. Toxicol. Pharmacol..

[131] Nakajima A., Nemoto K., Ohizumi Y. (2020). An evaluation of the genotoxicity and subchronic toxicity of the peel extract of Ponkan cultivar ‘Ohta ponkan’ (*Citrus reticulata* Blanco) that is rich in nobiletin and tangeretin with anti-dementia activity. Regul. Toxicol. Pharmacol..

[132] Li P., Wu H., Wang Y., Peng W., Su W. (2020). Toxicological evaluation of naringin: Acute, subchronic, and chronic toxicity in Beagle dogs. Regul. Toxicol. Pharmacol..

[133] Cohen S.M., Eisenbrand G., Fukushima S., Gooderham N.J., Guengerich F.P., Hecht S.S., Rietjens I.M.C.M., Bastaki M., Davidsen J.M., Harman C.L. (2019). FEMA GRAS assessment of natural flavor complexes: Citrus-derived flavoring ingredients. Food Chem. Toxicol..

[134] Ravichandran C., Badgujar P.C., Gundev P., Upadhyay A. (2018). Review of toxicological assessment of d-limonene, a food and cosmetics additive. Food Chem. Toxicol..

